# Mapping of Spatiotemporal Auricular Electrophysiological Signals Reveals Human Biometric Clusters

**DOI:** 10.1002/adhm.202201404

**Published:** 2022-10-31

**Authors:** Qingyun Huang, Cong Wu, Senlin Hou, Kuanming Yao, Hui Sun, Yufan Wang, Yikai Chen, Junhui Law, Mingxiao Yang, Ho‐yin Chan, Vellaisamy A. L. Roy, Yuliang Zhao, Dong Wang, Enming Song, Xinge Yu, Lixing Lao, Yu Sun, Wen Jung Li

**Affiliations:** ^1^ Department of Mechanical Engineering City University of Hong Kong Hong Kong 999077 P. R. China; ^2^ Department of Industrial Engineering and Management School of Mechanical Engineering Shanghai Jiao Tong University Shanghai 200240 P. R. China; ^3^ Hong Kong Centre for Cerebro‐cardiovascular Health Engineering (COCHE) Hong Kong Science Park New Territories Hong Kong 999077 P. R. China; ^4^ Department of Biomedical Engineering City University of Hong Kong Hong Kong 999077 P. R. China; ^5^ Department of Mechanical and Industrial Engineering University of Toronto Toronto M5S 3G8 Canada; ^6^ Bendheim Integrative Medicine Center Memorial Sloan Kettering Cancer Center New York NY 10065 USA; ^7^ James Watt School of Engineering University of Glasgow Glasgow G12 8QQ UK; ^8^ School of Control Engineering Northeastern University at Qinhuangdao Qinhuangdao 066004 P. R. China; ^9^ Shanghai Frontiers Science Research Base of Intelligent Optoelectronics and Perception Institute of Optoelectronics Fudan University Shanghai 200438 P. R. China; ^10^ Virginia University of Integrative Medicine Vienna VA 22182 USA

**Keywords:** full‐auricle electrophysiological monitoring, graphene‐based 3D electrodes, human biometric clusters, machine learning, personalized healthcare sensors

## Abstract

Underneath the ear skin there are rich vascular network and sensory nerve branches. Hence, the 3D mapping of auricular electrophysiological signals can provide new biomedical perspectives. However, it is still extremely challenging for current sensing techniques to cover the entire ultra‐curved auricle. Here, a 3D graphene‐based ear‐conformable sensing device with embedded and distributed 3D electrodes for full‐auricle physiological monitoring is reported. As a proof‐of‐concept, spatiotemporal auricular electrical skin resistance (AESR) mapping is demonstrated for the first time, and human subject‐specific AESR distributions are observed. From the data of more than 30 ears (both right and left ears), the auricular region‐specific AESR changes after cycling exercise are observed in 98% of the tests and are clustered into four groups via machine learning‐based data analyses. Correlations of AESR with heart rate and blood pressure are also studied. This 3D electronic platform and AESR‐based biometrical findings show promising biomedical applications.

## Graphical abstract

A new 3D contoured graphene‐based sensing array is used to map real‐time spatiotemporal auricular electrical skin resistance (AESR) signals. Through machine learning‐based data analysis, the human subject‐specific AESR distributions and auricular region‐specific AESR changes after cycling exercise are observed from the signals of more than 30 ears, which reveal the human biometric clusters for the first time.

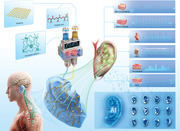

## 1 Introduction

Intelligent wearable electronics have become widespread with the advancements in material science, manufacturing technologies, and data science, for diverse healthcare applications involving physiological signal monitoring,^[^ ^1^, ^2^, ^3^, ^4^, ^5^, ^6^, ^7^, ^8^, ^9^, ^10^ ^]^ including electrocardiography (ECG), electroencephalography (EEG), heart rate (HR), blood pressure (BP), and body temperature. These vital physiological signs carrying various biophysical or biochemical information from human bodies are critical for pursuing long‐term and large‐scale monitoring for clinical diagnosis of diverse diseases.^[^ ^11^, ^12^ ^]^ Many electronic devices are worn on wrists, fingers, head, or legs record signals at single locations; however, signals measured at various spatial locations can provide additional dimension of crucial physiological information. For instance, full‐scale EEG monitoring with high‐density electrode arrays enables recording of electrical activity in multiple positions across the brain with complementary information provided during functional magnetic resonance imaging,^[^ ^13^, ^14^, ^15^ ^]^ and spatiotemporal cardiac measurements of ECG, pH, and temperature by integumentary membranes with conformable electrode arrays enables 3D mapping of epicardial signals.^[^ ^16^ ^]^

3D electronics developed for multi‐modal bio‐sensing with spatial resolution is receiving increasingly more attention recently due to the extremely complex surfaces of some human organs which have convex and concave shapes with various curvature radii.^[^ ^17^, ^18^ ^]^ Currently, 2D membrane‐based flexible and stretchable electronic devices can attach directly onto curved skin surfaces to monitor various physiological signals.^[^ ^19^, ^20^, ^21^, ^22^ ^]^ However, it is still very challenging to conformably cover the irregular 3D surfaces; in addition, the mass production of membrane‐based stretchable sensing devices is still a significant obstacle owing to the typically complicated and time‐consuming fabrication processes.^[^ ^23^ ^]^ For example, the human ear has very complex outer surfaces, which provides diverse physiological signals for health monitoring,^[^ ^24^, ^25^, ^26^, ^27^, ^28^, ^29^, ^30^, ^31^ ^]^ such as oxygen saturation, pulse, EEG, and body temperature. Underneath the auricular skin, a complicated nerve network involving branches of the greater auricular nerve, lesser occipital nerve, auriculotemporal nerve, facial nerve, and vagus nerve, as well as a vessel network involving branches of the superficial temporal artery and posterior auricular artery with varying sizes of diameters are formed across the auricles.^[^ ^32^, ^33^ ^]^ This special and intricate subcutaneous structure may also provide a wealth of physiological information varying with regions, such as BP and EEG. However, most current ear‐worn sensing devices with earplug‐like or clip‐like structures mainly focus on collecting data at a single location such as ear canal, earlobe, and antihelix. Thus, only temporal signal recording in specific region can be acquired and spatial‐level characterization is missing. For instance, auricular electrical skin resistance (AESR) signal has been used for diagnosing various human diseases, such as hepatic disorders,^[^ ^34^ ^]^ hip injury,^[^ ^35^ ^]^ breast cancer,^[^ ^36^ ^]^ type‐2 diabetes mellitus,^[^ ^37^ ^]^ and metabolic syndrome,^[^ ^38^ ^]^ and the pencil‐like commercial auricular detecting tools, which are primarily single‐probe electrical detectors with a rigid metal probe (such as Pointer Excel II, Lhasa OMS Inc., Weymouth, MA, USA), can measure point‐by‐point cutaneous conductance levels by manually moving the probe over the auricular skin surface. But the acquired signal is extremely sensitive to applied pressure, leading to low measurement repeatability. Thus, there is an urgent need for a conformable auricular sensing device that can provide a full‐auricle measurement of physiological signals to investigate their spatiotemporal characteristics.

**Figure 1:**
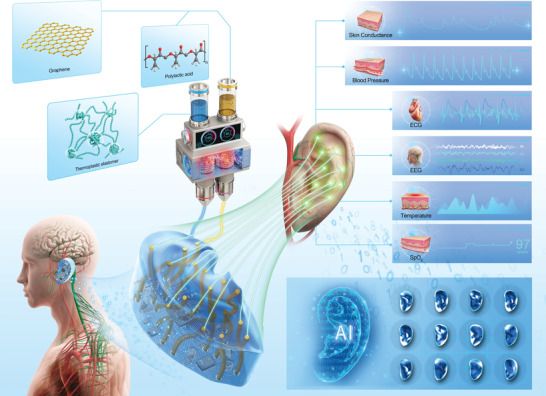
Schematic illustration of the 3D‐PAS monitoring platform and AI‐powered data analysis for promising biomedical and healthcare applications.

Here, we present the design and development of graphene‐based distributed 3D sensing electrodes for the acquisition of spatiotemporal physiological signals of the human auricles. A novel shape‐conformable personalized auricular sensor (3D‐PAS) has been demonstrated to enable real‐time electrophysiological signals mapping across the entire auricle (**Figure** **1**). This platform, with printable and programmable electrode threads, offers both conformable sensing interfaces with the curved auricular skin and 3D electrical interconnects. The entire sensor prototyping procedure, including human‐specific auricular shape acquisition, 3D electrode pathways design, and one‐step multi‐material 3D printing, is presented in this paper. Mechanical analysis of the skin surface curvature‐dependent electrode sensing area design was also performed. As a proof‐of‐concept, simultaneous measurement of electrical skin resistance which may reflect the subcutaneous biological conditions and vascular or neural activities^[^ ^39^, ^40^, ^41^, ^42^, ^43^, ^44^ ^]^ at multiple auricular points (APs) was demonstrated. In addition, an AESR contour is generated for 3D AESR mapping. For the first time, subject‐specific AESR distributions and auricular region‐specific AESR changes following physical exercise are demonstrated with unsupervised machine learning techniques. Finally, the correlations of AESR with HR and BP signals were also studied. These results provide potentially a universal platform and methodology for biomedical applications based on physiological signals of the auricles.

## 2 Results

### 2.1 Design and Fabrication

**Figure 2:**
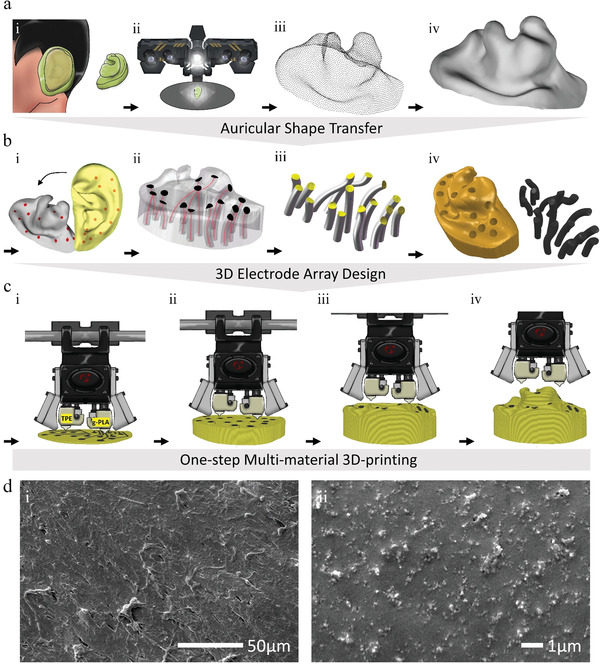
Diagram of the 3D‐PAS design and fabrication process. a) Illustration of human‐specific auricular skin‐shape acquisition and transfer: (i) Outer ear impression molding; (ii) 3D scanning of the auricular mold; (iii) point cloud generation; (iv) CAD modeling of the auricular mold. b) Illustration of 3D electrodes layout design: (i) APs locating; (ii) 3D electrode‐pathways embedding; (iii) geometric parameters scaling of 3D electrodes to achieve the same sensing area; (iv) sensor‐prototype assembling with 3D electrodes and the auricular mold. c) Illustrations of one‐step sensor prototyping: (i)–(iv) Sequential snapshots of 3D printing process integrating flexible TPE and conductive g‐PLA materials. d) SEM image of the printable (i) TPE material and (ii) g‐PLA material.

The entire fabrication process included three steps. First, geometrical information on the human‐specific outer ear structure was acquired and transferred (**Figure** **2**a). This began with the creation of a solid, 3D ear impression that shaped conformably with the auricle, whose surface curvature varies greatly between individuals and possesses a complex geometry. As shown in Figure 2a[i], medical‐grade molding polymers were evenly mixed and then filled into the entire outer ear, resulting in a personalized auricular mold. Structural light‐based 3D scanning (Figure 2a[ii]) was utilized on the auricular mold to generate a point cloud (Figure 2a[iii]), which was later merged into a solid 3D auricular mold geometry (Figure 2a[iv]) in a 1:1 scaled form. Second, a geometric layout for the 3D electrode pathways was designed inside the acquired auricular mold model (Figure 2b). Multiple electrodes were located at specific APs (Figure 2b[i]), where spatially separated pathways were extended across the auricular mold model accordingly and embedded inside with a tortuous layout to serve as 3D interconnects (Figure 2b[ii]). Here, the sensing areas of electrodes at multiple APs with varied surface curvatures were fixed to the same by scaling the geometric parameters (such as cross sections, diameter, and orientation) of the 3D pathways (Figure 2b[iii]). Third, the sensor was one‐step prototyped (Figure 2c). A well‐designed auricular mold prototype incorporated with multiple electrode pathways, defining the overall format of the sensor, was subsequently rendered by a commercial dual‐nozzle 3D printer integrating both a flexible elastomer and conductive graphene‐enhanced polylactide (g‐PLA) (Figure 2c[i–iv]). The surface topographies of these two materials are characterized by scanning electron microscopy (SEM) as shown in Figure 2d. The use of this materials combination provides good printability, comfortableness, and superior conductivity. The result of these steps was the creation of a 3D conformable and mechanically stable electrode‐skin sensing interface. The hardware control units were then connected to the printed electrode channels for data acquisition. Herein, we delivered a universal strategy involving a wearable 3D‐PAS device that offers a platform for geometrically integrating 3D‐distributed and conformable electrodes to achieve simultaneous multi‐region AESR collection in real time across the entire auricle.

### 2.2 Mechanical Analysis

**Figure 3:**
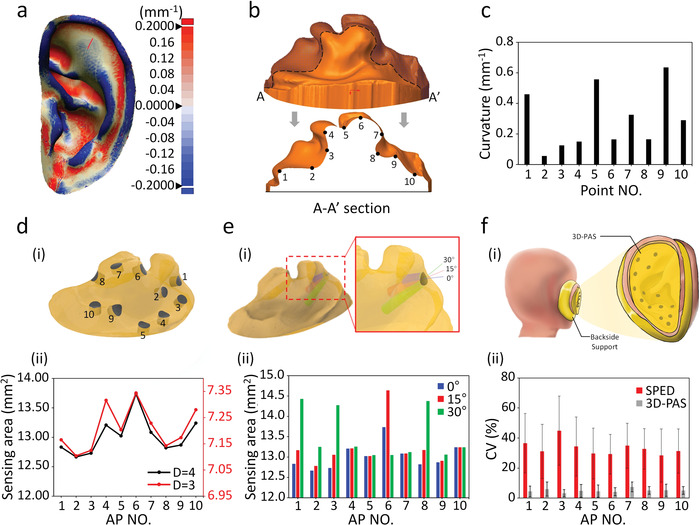
Mechanical analysis of the 3D‐PAS. a) Overall curvature distribution across the entire human auricle. b) Illustration of A–A′ cross section of 3D auricular mold. c) Curvature comparison at multiple points located along the cross‐sectional curve. d) Comparison of the electrode sensing area at multiple AP locations with varying surface curvatures: (i) Illustration of cylindrical electrode pathways with the determined diameter across spatially distributed APs; (ii) Sensing area comparison between multiple electrodes with pathway diameter *D* = 3 and 4 mm. e) Comparison of the electrode sensing area with different pathway orientations at each AP location: (i) Illustration of electrode pathways with the same size (3 mm diameter) across the AP with different orientations (blue: normal to surface, red: 15° from normal, green: 30° from normal); (ii) Sensing area comparison between different electrode pathway orientations at multiple AP locations. f) Analysis of AESR measurement repeatability: (i) Illustration of the mechanically stable sensing interface between the 3D‐PAS and auricular skin; (ii) Comparison of AESR measurement CV between a commercial single‐probe electrical detector and the 3D‐PAS.

A critical feature of this device is that it can be conformably shaped to the entire curved auricle and geometrically configured with multiple 3D electrodes with a profile‐controlled layout. The auricle of the ear, one of the smallest functional human organs, forms an ultra‐curved skin surface (**Figure** **3**a). This curvature varies not only within an auricular region but also across individual subjects, and this is also reflected in the geometry of the auricular mold. From one of the cross sections of the auricular mold (Figure 3b), it can be observed that the curvatures of ten points located along the cross‐sectional curve differ up to tenfold (Figure 3c). As a result, cylindrical electrode pathways with the same diameter across multiple APs with different surface curvatures and orientations will generate widely varying sensing areas (i.e., contact area) (Figure 3d,e), which can cause great deviations in contact resistance. Therefore, once the cross sections of the electrode pathways were determined, their geometric parameters (diameter, orientation, etc.) can be scaled point‐by‐point to achieve a consistent sensing area. Furthermore, to provide essential support from the backside of ear auricle, another ear mold was made and worn with the 3D sensor to fix the ear auricle between them without any observable mechanical shifting, which provides a 3D conformable and stable interface between the device and the entire auricle (Figure 3f[i]). As a result, three repeated AESR measurements on each of human subjects demonstrated excellent repeatability of the sensor with only a 4.9% average coefficient of variation (CV) among all testing points (Figure 3f[ii]). However, a commercial single‐probe electrical detector had a much larger CV of ≈35%, which was mainly caused by the significant deviation from operating pressure when moving across the auricle manually and freely without quantitative force feedback (Figure S1, Supporting Information). Here, an overall surface curvature‐dependent geometric design for the electrodes is implemented to ensure both conformability and consistency for the 3D AESR sensing interface.

### 2.3 Spatial AESR Mapping

**Figure 4:**
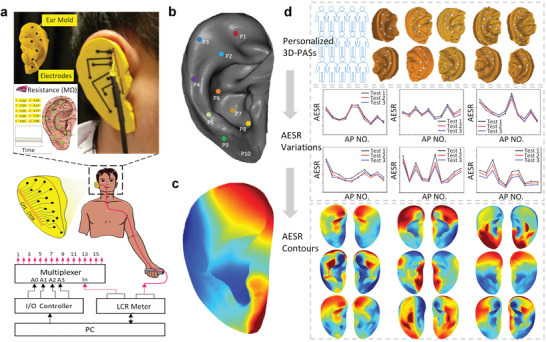
AESR mapping across the entire auricle. a) Illustration of AESR signal collection by the ear‐worn 3D‐PAS with a user interface and electrical control unit for multiplexed data acquisition. b) Spatial distribution of AP locations across the entire auricle. c) 3D AESR contour generation for visualizing the overall AESR distribution across the entire auricle. d) Highly consistent AESR variations across all tested APs and ASER contours collected from a large number of human subjects (60 ears) by fabricated personalized 3D‐PASs.

To demonstrate simultaneous multi‐region AESR monitoring with the 3D‐PAS, the positions of multiple testing points were first located (**Figure** **4**b). For each ear, a specific 3D‐PAS device was prototyped by the abovementioned procedures for AESR signal collection with a data acquisition unit. The whole electrical loop includes a sensor, the auricular skin, body, and LCR meter, where the circuit logically switches between the multiple electrodes with a multiplexer that prevents channel signal interference (Figure 4a). The AESRs were directly read from a self‐developed user interface in real time, which also marked all AP locations and highlighted the AP with the lowest AESR for further analysis; this can help systematically screen potential patients with abnormal AESR signals and advance auricular diagnoses. The AESR signals were stably collected across several minutes of monitoring (Figure S2a, Supporting Information). The variation in AESR levels across all testing APs in each measurement was drawn as a trend line (Figure S2b, Supporting Information), which maintains high consistency among multiple tests indicating excellent repeatability of measurement. Furthermore, based on the AESR data spatially mapped at the scattered APs, a 3D AESR contour with both geometric and spatiotemporal signal information was generated for the first time by a numerical interpolation algorithm to visualize the overall AESR distribution across the entire auricle (Figure 4c). By this way, a group of personalized 3D‐PASs was designed to collect different AESR variations across all tested APs and then generate various AESR contours from 60 ears (both right and left ears) (Figure 4d). This universal methodological route for 3D AESR mapping can be used to acquire quantifiable data for further study.

### 2.4 Human‐Specific AESR Distribution

**Figure 5:**
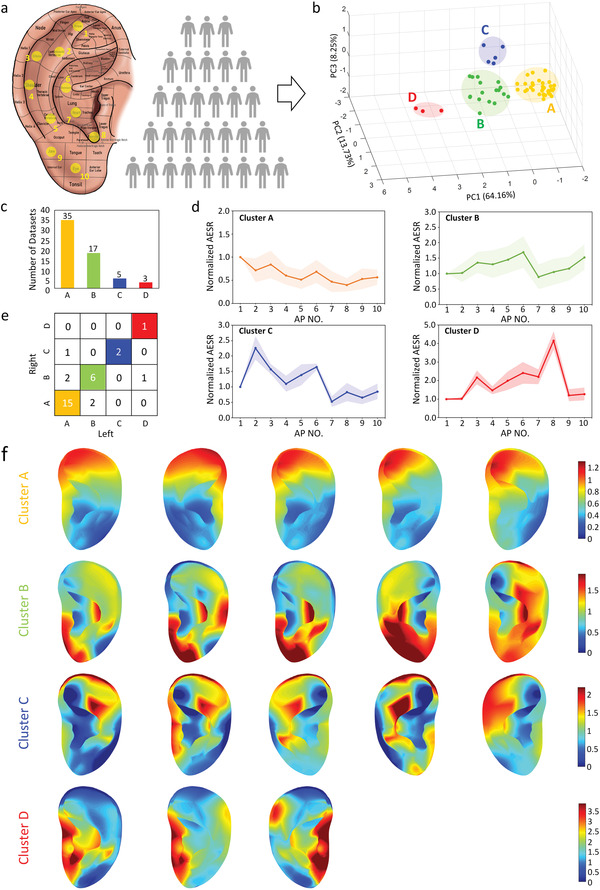
Human‐specific AESR distribution analysis. a) Schematic illustration of ten AP locations for all human auricles. b) *K*‐means clustering result for the AESR datasets collected from all 60 ears shown on a 3D scatter plot (each point denotes one AESR dataset from a single ear after PCA). c) Distribution of the total ear subject number in each cluster. d) Specific AESR variation trend lines across all tested APs with shaded error bands for each cluster. e) Matching results of both the left‐ and right‐ear datasets from each human subject after *K*‐means clustering (horizontal: cluster label of the left ear, vertical: cluster label of the right ear; diagonal numbers denote the number of subjects for whom both the left and right AESR datasets were classified to the same cluster (A, B, C or D). f) 3D AESR contours visualization for specific AESR distribution of ear subjects from each cluster (five representative subjects are selected in clusters A and B).

Using 3D‐PAS tools, comprehensive studies on the characteristics of the distribution of AESR among a human population were performed. Here, personalized devices were prototyped for both the left and right ears of 30 human subjects. In this paper, ten APs across the entire auricle were selected and located on all ear subjects with a proportionally scaled layout (**Figure** **5**a). After placing the 3D‐PAS into the ear, AESR signals were collected with the same procedures from all subjects, followed by data normalization. The AESR distribution across each ear was then characterized as a trend line which is drawn by connecting all the normalized AESRs of ten APs and packaged as a single dataset (i.e., a matrix of 1 by 10). Unsupervised machine learning techniques, including principal component analysis (PCA) and *K*‐means clustering, were performed on the AESR datasets collected from all 60 ears (i.e., a pair ear per subject). First, PCA achieved 86.1% of the total explained variance (EV) from raw data with three principal components (PCs) (Figure S3a, Supporting Information). Then, *K*‐means clustering algorithm was performed, here we calculated the sum of the square error (SSE) and use the elbow method to find that 4 was the optimal value of cluster number *K* that yielded the greatest cluster‐to‐cluster separation and best within‐cluster gathering (Figure S3b, Supporting Information); we also achieved a high silhouette score (S‐score) of 0.76 (Figure S3c, Supporting Information). This result indicates that all AESR datasets were clearly classified into four clusters, while each cluster showed a specific AESR distribution across the ten APs. Accordingly, four clusters of 3D scattered points were acquired by PCA dimensionality reduction, and were plotted to visualize the clustering result (Figure 5b). Four trend lines with shaded error bands (Figure 5d) also depicted the specific AESR distribution of clusters A–D, which included 35, 17, 5, and 3 ears, respectively (Figure 5c). By matching the clustered datasets with the subject labels, it was observed that 80% of the tested subjects (i.e., 24/30) had their left‐ and right‐ear AESR distribution classified into the same cluster (Figure 5e). This preliminarily indicates that AESR distribution information of both auricles is highly similar. Furthermore, the 3D AESR contours of all ears were generated; the clustering result can also be characterized by these contours, which directly illustrate the high consistency in the 3D AESR distribution among within‐cluster subjects as well as the large variability among across‐cluster subjects (Figure 5f and Figure S4, Supporting Information). Here, we demonstrated a novel biometrical tool for human population classification, which shows great potential to serve as a new form of biological marker.

### 2.5 Region‐Specific AESR Change after Exercise

**Figure 6:**
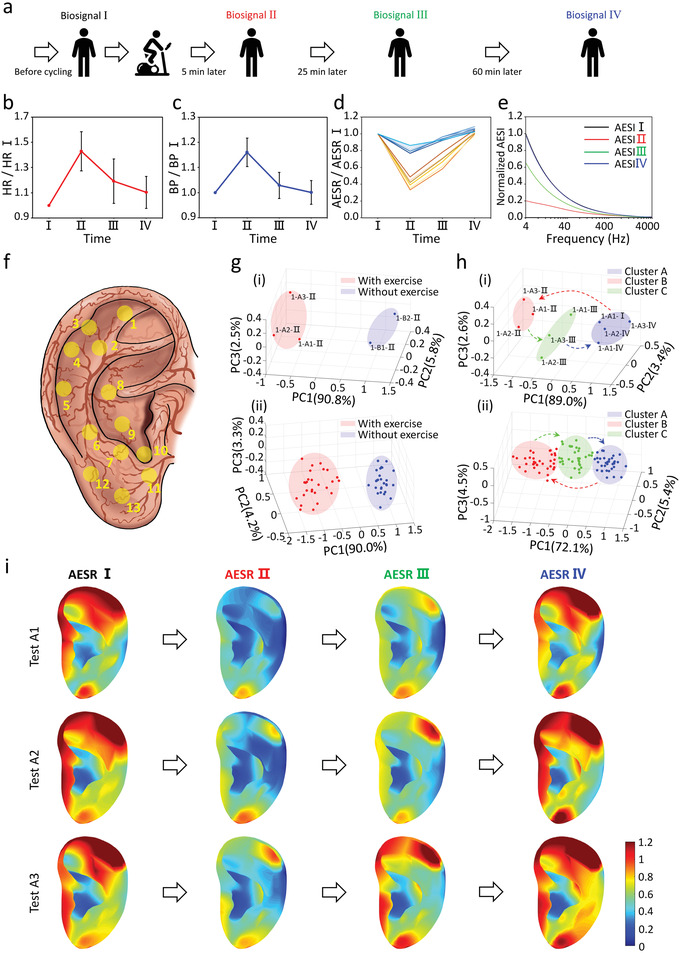
Analysis of after‐exercise region‐specific AESE change. a) Schematic illustration of biosignals (HR, BP, AESR, and AESI) collection in four sequential periods in each cycling test. b) HR, c) BP, d) AESR (different color represents impedance measured at different channels: warm color curves are the AESR levels at AP1‐6, cold color curves are the AESR levels at AP7‐13), and e) AESI changes after fixed‐intensity cycling exercise. f) Locations of the studied 13 APs for all human ear auricles. g) 3D scatter plots showing *K*‐means clustering results for the AESR datasets collected from the cycling/control tests for (i) an individual volunteer and (ii) all 17 volunteers (“1‐A1‐II” denotes the AESR dataset collected in the period II in the first cycling test from volunteer 1). h) 3D scatter plots showing *K*‐means clustering results for the AESR datasets collected in each of the four periods from the three cycling tests for (i) an individual volunteer and (ii) all 17 volunteers. i) 3D AESR contours illustrating overall AESR distribution changes across all four periods in the cycling tests (one representative volunteer is selected).

Using 3D‐PAS tools, the study on individual AESR change when subjected to physiological stimuli was investigated for the first time. Stationary cycling, a popular physical exercise worldwide, causes a whole‐body physiological response,^[^ ^45^ ^]^ including an increase HR to meet the muscles’ demand for oxygen and a surge in BP with the increased availability of vasodilatory mediators. To explore the effects of physical exercise on AESR levels, 17 volunteers participated in a study to repeat stationary cycling exercises three times at a fixed intensity (i.e., test A1–A3). Here, three additional APs (i.e., AP3, 7, and 11) were added to the ten previously studied APs (**Figure** **6**f). All tests followed the same procedures. During each test, biosignals were collected at four fixed periods (i.e., period I–IV) before and after cycling (Figure 6a). Two additional tests of controls were also performed without cycling (i.e., test B1 and B2), where the AESR signals were collected in the same four periods. Meanwhile, HR and BP were recorded by a commercial smart watch and a cuff‐style BP monitor, respectively. 5 min after the completion of the exercise, it was observed that HR and BP increased by 42.9% and 16.0%, respectively, on average over all tests (Figure 6b,c). Correspondingly, the AESR levels at AP1‐6 dramatically dropped by 60.8%, 66.8%, 55.4%, 64.9%, 57.8%, and 51.3% on average, while those at AP7‐13 dropped by less than 23.5% (Figure 6d and Figure S5, Supporting Information). In 98% of total 51 individual cycling tests, auricular region‐specific AESR changes were observed under the stimuli of physical exercise. Comparatively, AESR levels showed no change across the four periods in all tests of controls (Figure S6, Supporting Information). Similar change of the auricular electrical skin impedance (AESI) scanning from 4 to 4000 Hz is also shown in Figure 6e and Figure S7, Supporting Information.

For each measurement, the AESRs collected at each of the 13 APs were point‐to‐point normalized by their initial AESRs (AESR I) and repackaged into a new dataset (i.e., matrix of 1 by 13) labeled as “Subject‐Test‐Period.” Unsupervised machine learning was then utilized to classify the datasets comprising the data from all five tests for each volunteer, including three datasets from the repeated cycling tests and two from the control tests, using the AESR II. Following PCA which achieved a 98.5% average total EV with three PCs (Figure S8a, Supporting Information), *K*‐means clustering was performed; the optimal value of cluster number *K* determined by the elbow method was 2 (Figure S8b, Supporting Information), while the S‐score was 0.88 on average (Figure S8c, Supporting Information), which indicates that the two clusters of datasets can be clearly classified. In the analysis for 94% (i.e., 16 out of 17) of the volunteers, datasets in these two clusters were exactly label‐matched to the AESR signals collected in cycling tests and control tests, respectively. A 3D scatter plot was shown to visualize the individual clustering results (Figure 6g[i] and Figure S8d, Supporting Information). Furthermore, similar PCA and clustering analyses were performed for the datasets comprising the data from all the tests on 17 volunteers. Following PCA, the total EV with three PCs was 88.3% (Figure S9a, Supporting Information), and similar to the prior analysis, two clusters (Figure 6g[ii]) were classified with *K* = 2 (Figure S9b, Supporting Information) and an S‐score of 0.85 (Figure S9c, Supporting Information).

Additionally, it was also observed that the AESR at periods III and IV of AP1‐6 gradually increased back to 67.7% and 100% of the initial value on average, demonstrating a highly consistent trend with HR and BP. Here, unsupervised machine learning was also used to classify the normalized datasets comprising the AESR data collected at the four periods across the three repeated cycling tests for each volunteer. First, PCA achieved 93.4% total EV on average with three PCs (Figure S10a, Supporting Information). Then *K*‐means clustering was performed; when *K* was determined as 3, analysis for datasets from 76.5% (i.e., 13 out of 17) of the volunteers obtained the result that, one dataset of “AESR II” together with three datasets of “AESR IV” were classified into cluster A, three datasets of “AESR II” were classified into cluster B, and three datasets of “AESR III” were classified into cluster C. A 3D scatter plot visualized this clustering result, in which three arrows were drawn to illustrate cluster‐point shifting and match the dynamic evolution of the whole‐body response (Figure 6h[i] and Figure S10b, Supporting Information). Furthermore, similar machine learning results were obtained for the AESR datasets collected from all cycling tests from the 17 volunteers; PCA yielded 82% total EV with three PCs (Figure S11a, Supporting Information), the elbow method yielded a *K* of 3 (Figure S11b, Supporting Information), the S‐score was 0.64 (Figure S11c, Supporting Information), and 85.1% of the datasets achieved the same label‐group matching result described in the above individual volunteer analysis (Figure 6h[ii]). Last, a highly consistent region‐specific color change across the four periods can be observed from the generated 3D AESR contours (Figure 6i and Figure S12, Supporting Information). Here, both data‐driven and vision‐driven analyses validate the auricular region‐specific AESR change as the response to physical exercise stimuli.

**Figure 7:**
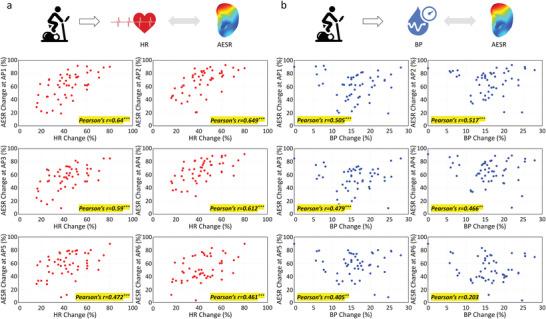
Correlation analysis of AESR with HR and BP. Scatter plots of changes in AESR at AP1‐6 versus changes in a) HR and b) BP after fixed‐intensity cycling exercise (Biosignals II collected in all cycling tests from 17 volunteers are used).

Furthermore, it was observed that AESR changes differed greatly test‐to‐test after fixed‐intensity exercise. This indicated the presence of individual body condition differences, which were also reflected in the changes in HR and BP signals. Scatter plots comparing the changes in AESR with the changes in HR and BP across all cycling tests from 17 volunteers are shown in **Figure** **7** and Figure S13, Supporting Information. Statistical analysis was performed on these biosignals point by point. It obtained that the Pearson correlation coefficient (Pearson's *r*) was 0.57 on average for the “AESR‐HR” comparison across AP1‐6, and was 0.49 on average for the “AESR‐BP” comparison across AP1‐4, indicating that AESR certainly correlates with HR and BP at most of the “active points” (AP1‐6). On the other hand, there is no correlation of AESR with HR and BP (*p* > 0.05 or Pearson's *r* < 0.4) at most of “inactive points” (AP7‐13).

## 3 Discussion

The design of the 3D‐PAS provides a 3D conformable sensing interface with multiple electrodes whose locations, layout, size, and density can be scalably altered by demand. In the future, electronic elements with functionalities such as signal conditioning, multiplexing, analog‐to‐digital conversion, wireless transmission, and power supply, can be embedded into the 3D AESR sensing device so that the sensor can become a ready‐to‐use wearable electronic device. In addition to AESR, other auricular physiological biosignals (such as BP, blood oxygen saturation, temperature, and hydration) can also be monitored and mapped through the strategy of sensor design and data analysis introduced in this paper. Furthermore, diverse physical stimulating functions (electrical, mechanical, thermal, etc.) can be developed to assist auricular therapies by applying alternative functional materials for 3D electrodes. Accordingly, multi‐modal sensing together with stimulation over the entire auricle would be quite promising for comprehensive health evaluation and management.

In this paper, the characteristics of the AESR distribution were investigated among 30 human subjects. The result is that four types of AESR distributions across all testing APs were clearly classified. Interestingly, it was observed that subjects in cluster A, B, and D have a recognizable age‐level difference (Figure S14, Supporting Information), which may reveal that the separation of these clusters can partially be explained by “age differences.” However, given the age‐level overlap for a few subjects from different clusters, additional hidden classification criteria may also exist. Therefore, more studies involving a larger cohort of subjects in the future may help in forming a comprehensive understanding of the underlying physical meaning for these interesting observations. Furthermore, within each cluster, the AESR distributions of all subjects were essentially similar with only minor differences between them. Meanwhile, the AESR datasets collected from repeated measurements on multiple subjects from the same cluster can also be clustered into individuals (Figure S15, Supporting Information). This indicates that each subject has a unique form of AESR distribution, which, in the vein of a “fingerprint,” can be called an “earprint.” Given the above analyses, AESR signals hold great potential to serve as a new form of biological marker, similar to BP or HR, for matching individuals with diverse body conditions.

In the cycling tests on 17 volunteers, much greater AESR changes were consistently observed after exercise at AP1‐6 than at AP7‐13, which may reflect the varying levels of hemodynamic and neural activity underneath skin in different auricular regions. The gradual increase in the AESRs back to their baseline levels is consistent with the evolution of the condition of the body during recovery after exercise. In the statistical analysis, the 95% confidence intervals (shown in Figure S16, Supporting Information) were calculated by using Bootstrap method (1000 samples) for AESR and HR/BP datasets correlation analysis, verifying that there exists the relationship between HR and AESR at AP1‐6, and between BP and AESR at AP1‐4. The fact that AESR didn't correlate with BP at AP5‐6 and that smaller Pearson's *r* was obtained for the “AESR‐BP” comparison than for the “AESR‐HR” comparison may have resulted primarily from the high inconsistency in the noncontinuous BP measurement with the commercial, cuff‐style BP monitor. Furthermore, ≈10% of all the datasets were abnormal, 80% of which were collected from only two individual volunteers who may differ from the others in body condition. On the other hand, the outer ear skin temperature was simultaneously collected during each test (Figure S17, Supporting Information), which does not show the similar region‐specific change with AESR data; this result indicates that temperature change is not a significant factor contributing to the AESR changes observed in this work. These findings provide quantitative evidence for auricular region‐specific AESR change under physiological stimuli. Thus, following further studies, AESR monitoring is promising for personalized healthcare applications.

AESR at specific ear auricle region has been an effective diagnostic tool for various diseases,^[^ ^34^, ^35^, ^36^, ^37^, ^38^ ^]^ and AESR distribution has been shown in this work to correlate to different body physiological conditions. Hence, AESR signals show great potential in serving as a new diagnostic indicator to supplement conventional diagnostic methods, especially for complicated diseases when conventional physiological signal measurement cannot provide definitive prognostics. However, much more studies are required to understand how the AESR distributions reflect different body physiological conditions and how AESR information can correlate and couple with conventional physiological signals with biomedical explanations. Here, our work is to demonstrate the first working 3D sensing device that enables big data collection of AESR signals for supporting further medical explorations.

Additionally, real‐time and continuous auricular electrical signals could be used in the future to track or modulate human neural responses, such as neuropathology monitoring of depression,^[^ ^46^ ^]^ management of chronic musculoskeletal pain,^[^ ^47^ ^]^ and sleeping monitoring.^[^ ^48^ ^]^ Furthermore, other types of physiological signal measurements (such as electroencephalogram, pulse oximetry, temperature, galvanic skin response, and electromyography) can also be integrated with the developed 3D AESR sensor with a sampling rate ranging from 10 to 1000 Hz to match existing and well‐accepted human physiological instrumentation methods. Distribution of these continuous auricular signals could be promising for future medical diagnostic applications.

In summary, a personalized wearable device incorporating distributed 3D electrodes for simultaneous, multi‐region AESR monitoring across the entire auricle was developed. Data analyses, including 3D contours for spatiotemporal AESR mapping and unsupervised machine learning for AESR datasets classification, reveal for the first time human‐specific AESR distribution, and the correlations of AESR spatiotemporal electrophysiological signals with HR and BP. This strategy of 3D electrodes design and full‐auricle sensing platform shows promising biomedical applications for spatiotemporal auricular physiological monitoring during daily activities.

## 4 Experimental Section

### Fabrication of the 3D‐PAS

The whole process started with the construction of personalized 3D auricular impressions by molding with medical‐grade polymers and catalyzers (Green Eco, DETAX), which were mixed at a ratio of 1:1 and then injected into the entire auricle as well as the backside with a syringe. After a few minutes, the mixture solidified, and the auricular mold was shaped. 3D scanning of the auricular mold was performed by a structural light‐based 3D scanner (Dual‐laser‐source, 0.001 mm resolution, Nanyangmengyang Machinery Co., Ltd, China) to generate a point cloud, which was then merged into a 3D solid model whose surface was further modified in computer‐aided design (CAD) software. Testing points were located with a proportionally scaled layout. The layout, orientation, and size of 3D electrode pathways were geometrically designed within the auricular mold in CAD software. A single sensor that incorporated the auricular mold and the 3D electrodes was then one‐step prototyped by a dual‐nozzle 3D printer (RAISE 3D PRO 2, USA) with optimal printing parameters (Table S1, Supporting Information), which integrated thermoplastic elastomer (TPE) (TPE‐85A, Esun Industrial Co., Ltd., China) and g‐PLA (BLACKMAGIC3D Company, USA) printable materials. The surface topographies of these two materials were characterized by SEM. The flexible TPE enabled good comfortableness in contact with the auricular skin and integrated well with the g‐PLA material, offering a superior conductivity of 1.7 S cm^−1^. The good printability of this materials combination was realized by optimizing the printing parameters (e.g., extruder temperature and extrusion rate). Last, commercial conductive gel (slowly drying gel, Jinxin Chemistry Industry Company Ltd, China) was thinly coated onto the printed electrode surfaces prior to measurement. Electrical circuit and cables were connected to the sensor by using simple electrical soldering iron heating process.

### Measurement Calibration

For each single electrode on the 3D‐PAS, the accuracy of the electrical resistance measurement was calibrated by a series of constant resistors ranging from 0.5 to 10 MΩ. The temperature coefficient of resistance was also characterized in a laboratory oven as around 2.6 Ω °C^−1^ over a range of room temperatures (25–35 °C), which was negligible relative to skin resistance (Figure S18, Supporting Information). Therefore, the 3D‐PAS could achieve good measurement accuracy, efficiency, visuality, and repeatability far beyond those of manually operated, commercial single‐probe electrical detectors.

### Mechanical Analysis

The distribution of the curvature of the entire auricle skin surface was acquired by the internal curvature analysis function built in CAD software, which also provided the curvature‐dependent sensing area for the conformable electrodes with predetermined locations and geometrical parameters. To verify if the contact and conductivity between the graphene electrodes of the 3D sensor and auricular skin was indeed stable, a thin layer of conductive gel (which was eventually applied to cover the 3D‐printed sensing graphene electrodes) was applied at specific locations (i.e., corresponding to the locations of the sensing electrodes in the actual 3D sensor) on the surface of 3D ear molds (i.e., molds without graphene electrodes). Some subjects were asked to wear these test molds and the results showed that, even with the possible motion artifacts and the effect of sweat, the thin layer of conductive gels could be accurately bonded and transferred to the corresponding locations on the ear auricles without any shifting when the ear molds were taken off from the subjects. These results indicated that the conductive gel used in this work had a significant and stronger adhesive bond to the ear skin than the mold material. And, when the gel was used in the actual 3D sensing molds (i.e., with the graphene electrodes), it showed a stronger bond to the graphene electrodes than the ear skin, that is, they would not adhere to the ear skin and would stay on the electrodes of the sensing molds when the sensing modes were removed from the subjects. Therefore, the graphene electrodes of the 3D sensor had a good contact and conductive interface with the subjects’ ear skin for high quality bio‐signal acquisition.

### Human Studies

In total, 30 volunteers participated in the AESR distribution study, and 17 volunteers participated in the cycling exercise tests. Before each measurement, medical alcohol pads were used to remove oil, sweat, and other impurities on the auricular skin surface followed by air drying. In the investigation of AESR change after physical exercise, each volunteer was asked to repeat three stationary cycling tests at a fixed intensity (7 km within 20 min) with at least a 48‐h interval on a fitness bike (Decathlon EB 500 SP, France) and two control tests without cycling during the same time slot on a separate day. For the individual tests, AESR, HR, and BP signals were recorded in four sequential periods: before exercise (period I) and 5 min (period II), 25 min (period III), and 60 min after the completion of the exercise (period IV). Room temperature and humidity were also monitored, demonstrating negligible fluctuations during the whole test.

### Ethical Declaration

All human cycling studies were implemented in accordance with the ethical guidelines and with the approval of the Human Subject Ethics Committee of City University of Hong Kong (Reference No.: 10‐2020‐19‐F). Written informed consent was obtained from all participants with the purpose and procedures of the study clearly explained.

### Data Acquisition

AESR was measured by the LCR‐meter function embedded in the impedance analyzer (HIOKI‐IM3570, Japan), with a 16‐channel multiplexer (ADG706) switching between multiple electrodes. Two‐probe measurement setup was used and all measuring electrodes share one common reference electrode which was connected to a subject's hand. HR was monitored by a commercial smart watch (HUAWEI WATCH GT 2 Pro, China). BP was measured by a cuff‐style commercial monitor (Omron HEM‐7320, Japan). Room temperature and humidity were measured by a commercial monitor (Jiandarenke COS‐03, China). Auricular skin temperature was measured by a commercial infrared thermal camera (Hikvision, China).

### Data Analysis—AESR normalization

In the investigation of AESR distribution among human population and generation of 3D AESR contours, AESR levels measured at all testing APs from each subject were normalized to those at AP1 for further analysis. In the investigation of AESR change under physical exercise conditions, including both cycling and control tests, the AESRs collected in periods I–IV at each AP were normalized to those in period I (i.e., initial value).

*3D AESR contour generation*: The normalized AESR signals collected at multiple APs and 3D coordinates of the point cloud acquired from auricular shape 3D‐scanning were input to perform natural neighbor interpolation for 3D scattered data in MATLAB, generating a 3D AESR contour with a continuous AESR gradient to visualize the overall spatial AESR distribution across the entire auricle.

*AESR dataset package*: Normalized AESRs collected at *N* APs in one single measurement were packaged into a single dataset, that is, a matrix of 1 by *N*.

*Unsupervised machine learning analysis*: All normalized AESR datasets collected from *M* tests were incorporated into an input matrix (*M* × *N*), which was later dimension‐reduced to an *M* × *3* matrix by PCA. Then, the *K*‐means clustering algorithm was utilized to classify the datasets in MATLAB, where the Euclidean distance and “*K*‐means++” clustering center initialization were used; the other parameters were set to their default values.

*Determination of K*: The optimal value of cluster number *K* was determined by the “elbow method,” that is, by selecting the elbow point in the SSE‐K plot. The SSE for each cluster was calculated as Equation (1) and the S‐score of each dataset was acquired as Equation (2).

(1) $$\mathrm{SSE}=\sum_{i=1}^{k}\sum_{x\in C_{i}}^{t}dist^{2}\left(m_{i},x\right)$$

where *x* was a data point in cluster *C_i_* and *m_i_* was the center point for cluster *C_i_*.

(2) $$s\left(i\right)=\left\{\begin{array}{cc} 1-a\left(i\right)/b\left(i\right), & \mathrm{if}\;\;a\left(i\right)<b\left(i\right)\\ 0, & \mathrm{if}\;\;a\left(i\right)=b\left(i\right)\\ b\left(i\right)/a\left(i\right)-1, & \mathrm{if}\;\;a\left(i\right)>b\left(i\right) \end{array}\right.$$

where $a\left(i\right)=\frac{1}{|C_{i}|-1}\sum_{j\in C_{i},i\neq j}d\left(i,j\right)$, $b\left(i\right)=\min_{k\neq i}\frac{1}{|C_{k}|}\sum_{j\in C_{k}}d\left(i,j\right)$, and i was a data point in cluster *C_i_*.

*Correlation analysis: p*‐values for significance testing and Pearson's *r* were acquired by SPSS software after excluding abnormal datasets; confidence intervals were calculated using Bootstrap method (1000 samples) by MATLAB software to verify the 95% confidence level for the true values.

## Conflict of Interest

The authors declare no conflict of interest.

## Author Contributions

Q.H. and C.W. contributed equally to this work. W.J.L., Q.H., and H.C. conceived the basic idea of this study and sensor design. W.J.L., Q.H., and C.W. designed the experiments. Q.H. performed the experiments. Q.H., W.J.L., H.C., X.Y., V.A.L.R., Y.C., Y.W., and Y.S. analyzed the data. M.Y. and L.L. provided support for medical related information and interpretation of data for this study. K.Y., J.L., H.S., and S.H. assisted in part of experiments and sensor fabrication process. Q.H., W.J.L., and C.W. co‐drafted this paper. X.Y., E.S., V.A.L.R., Y.Z., D.W., and Y.S. provided critical technical advice for this study. W.J.L. and H.C. supervised the project.

## Supporting information

Supporting Information

## Data Availability

The data that support the findings of this study are available in the supplementary material of this article.
