# Supplementary material for: Mapping of Spatiotemporal Auricular Electrophysiological Signals Reveals Human Biometric Clusters
Source: Adv Healthc Mater. 2022 Oct 31;11(23):2201404. doi: 10.1002/adhm.202201404 (PMC11469291; doi:10.1002/adhm.202201404)
Supplement: Supplementary file 1 — Supporting Information [file ADHM-11-2201404-s001.pdf]

Supplementary Materials for

Mapping of Spatiotemporal Auricular Electrophysiological Signals Reveals Human Biometric Clusters

Qingyun Huang[1,2†], Cong Wu[1,3†], Senlin Hou[1], Kuanming Yao[4], Hui Sun[1], Yufan Wang[2], Yikai Chen[2], Junhui Law[5], Mingxiao Yang[6], Ho-yin Chan[1]*, Vellaisamy A. L. Roy[7], Yuliang Zhao[8], Dong Wang[2], Enming Song[9], Xinge Yu[3,4], Lixing Lao[10], Yu Sun[5], Wen Jung Li[1,3]*

Correspondence to:

wenjli@cityu.edu.hk (W. J. Li)

This PDF file includes:

Figs. S1 to S18

Table S1

List of contents:

Figure S1. Electrical skin conductance measurement results from a commercial single-probe electrical detector.

Figure S2. AESR measurement results from 3D-PAS.

Figure S3. Unsupervised machine learning results for the datasets of human-specific AESR distribution collected from 30 human subjects in this study.

Figure S4. Clustered AESR contours in 3D for 30 human subjects.

Figure S5. Normalized AESRs collected at 13 auricular points in the cycling test.

Figure S6. The average change of AESR for the control group in the cycling test.

Figure S7. Normalized AESIs collected at 13 auricular points in the cycling test."

Figure S8. Unsupervised machine learning results for the AESR datasets collected from the 17 individual volunteers in the cycling and control tests.

Figure S9. Unsupervised machine learning results for the AESR datasets collected from all the 17 volunteers in the cycling and control tests.

Figure S10. Unsupervised machine learning results for the AESR datasets collected from the 17 individual volunteers during four periods in the cycling test.

Figure S11. Unsupervised machine learning results for the AESR datasets collected from all the 17 volunteers during four periods in the cycling test.

Figure S12. 3D AESR contours from the 17 individual volunteers change during four periods in the cycling test.

Figure S13. Correlation analysis of AESR with HR and BP.

Figure S14. Statistical result of age for 30 human subjects with 4 clusters.

Figure S15. Unsupervised machine learning results for the AESR datasets collected from 3 human subjects in cluster B.

Figure S16. Estimated correlation coefficient distribution histogram with 95% confidence intervals.

Figure S17. Demonstration of auricular skin temperature.

Figure S18. Temperature coefficient of resistance measurement for the printable g-PLA filament.

Table S1. Optimal printing parameters for 3D-PAS prototyping.

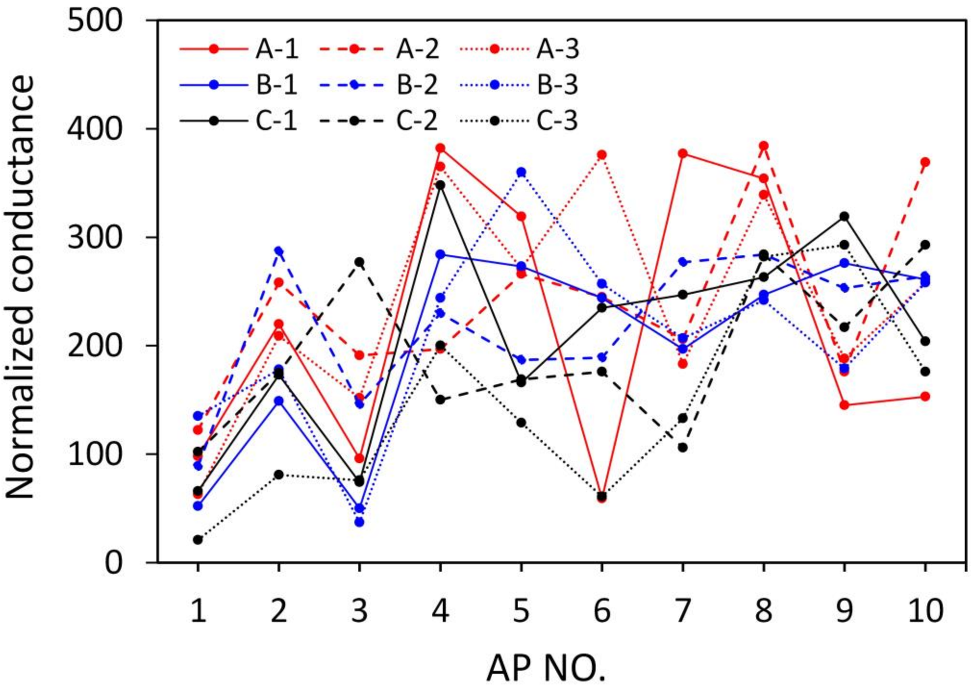

Figure S1. Electrical skin conductance measurements result from a commercial SPED operated by three testers (A, B & C). All three measurements (1, 2 & 3) were obtained from the same human ear auricle.

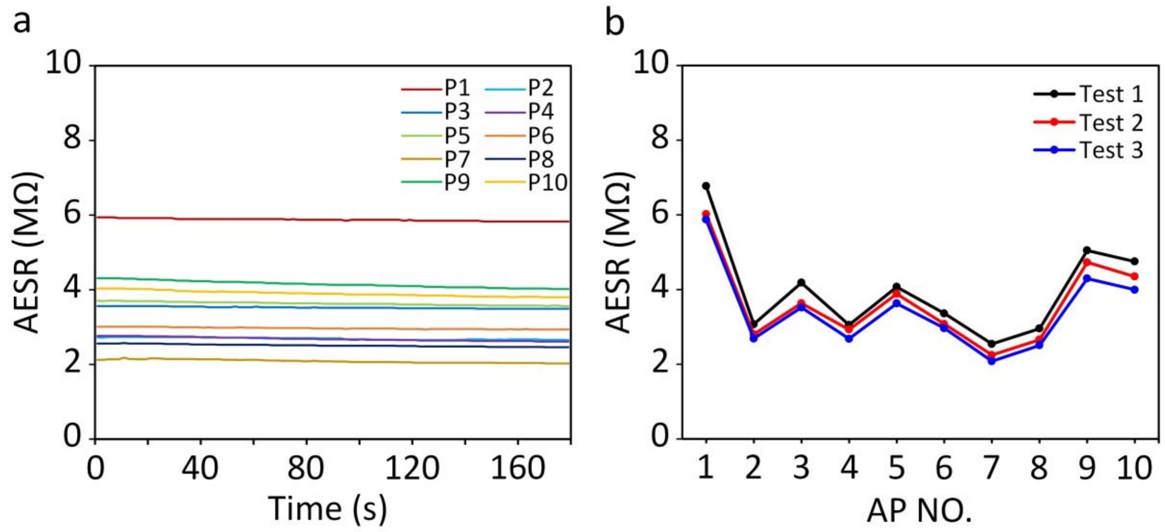

Figure S2. AESR measurement result from 3D-PAS. **(a)** Simultaneously stable AESR signal collection at multiple APs in real time; **(b)** Highly consistent AESR variation across all tested APs between repeatable tests.

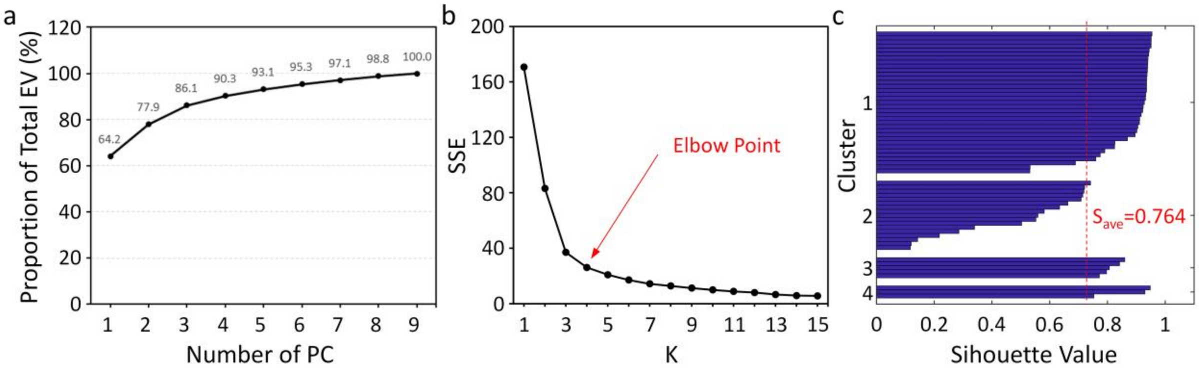

Figure S3. Unsupervised machine learning results for the AESR datasets collected from 30 human subjects in the study on human-specific AESR distributions. **(a)** Proportions of total explained variance from the raw datasets following PCA with different numbers of PCs picked in order of decreasing variance contribution; **(b)** SSE changes with different values of cluster number K in the K-means clustering process; **(c)** S-score bar chart for the datasets in each cluster in the K-means clustering process with K=4.

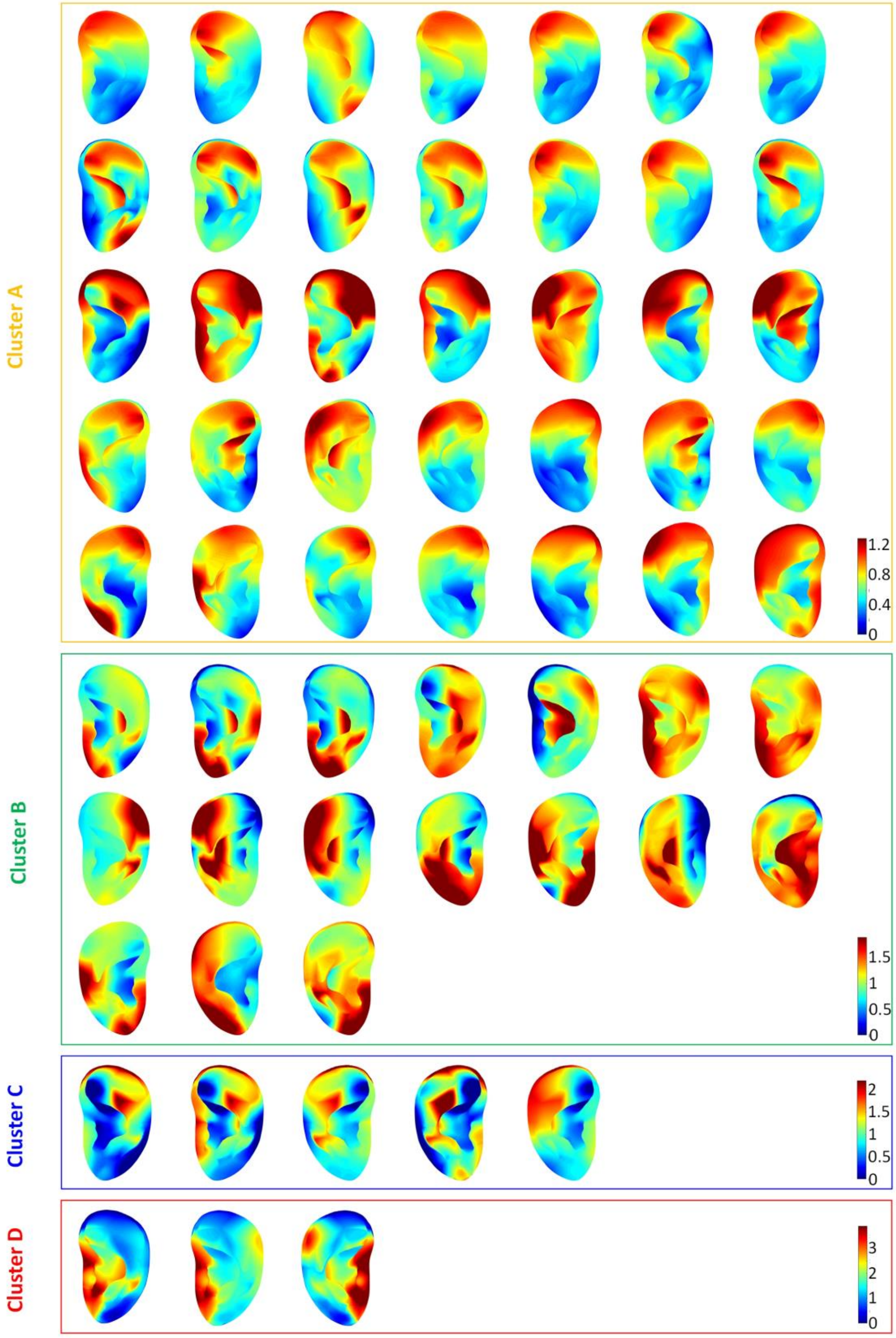

Figure S4. Clustered 3D AESR contours from 30 human subjects (both right and left ears).

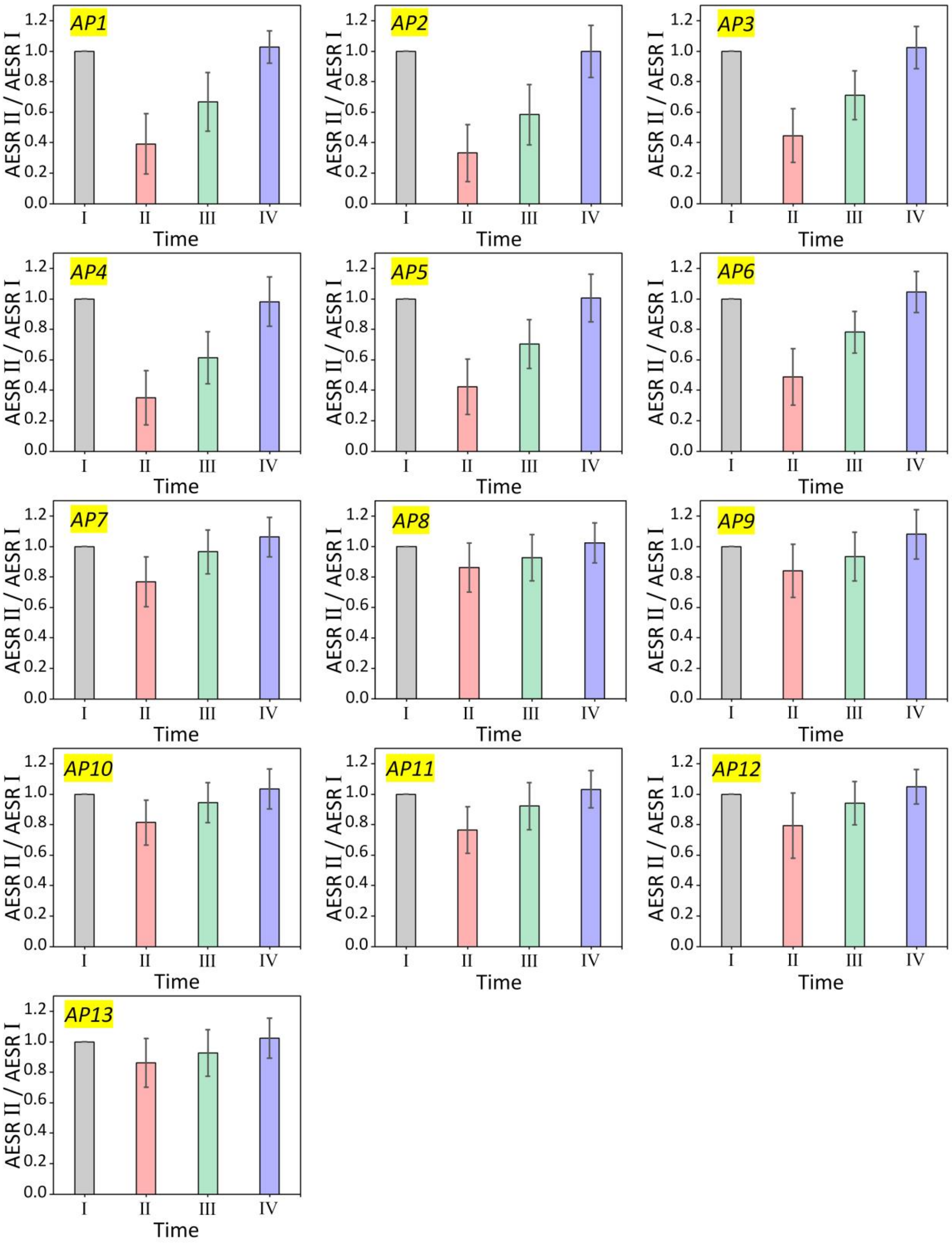

Figure. S5. Normalized AESRs collected at AP1-13 across the four periods during all the cycling tests from 17 volunteers.

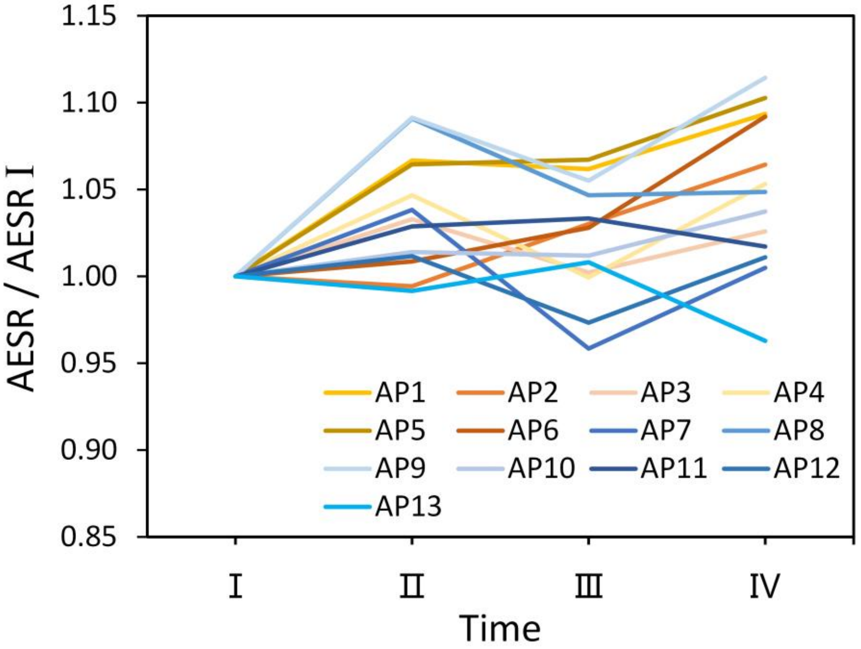

Figure. S6. AESR average changes across the four periods during all tests of control from all 17 volunteers.

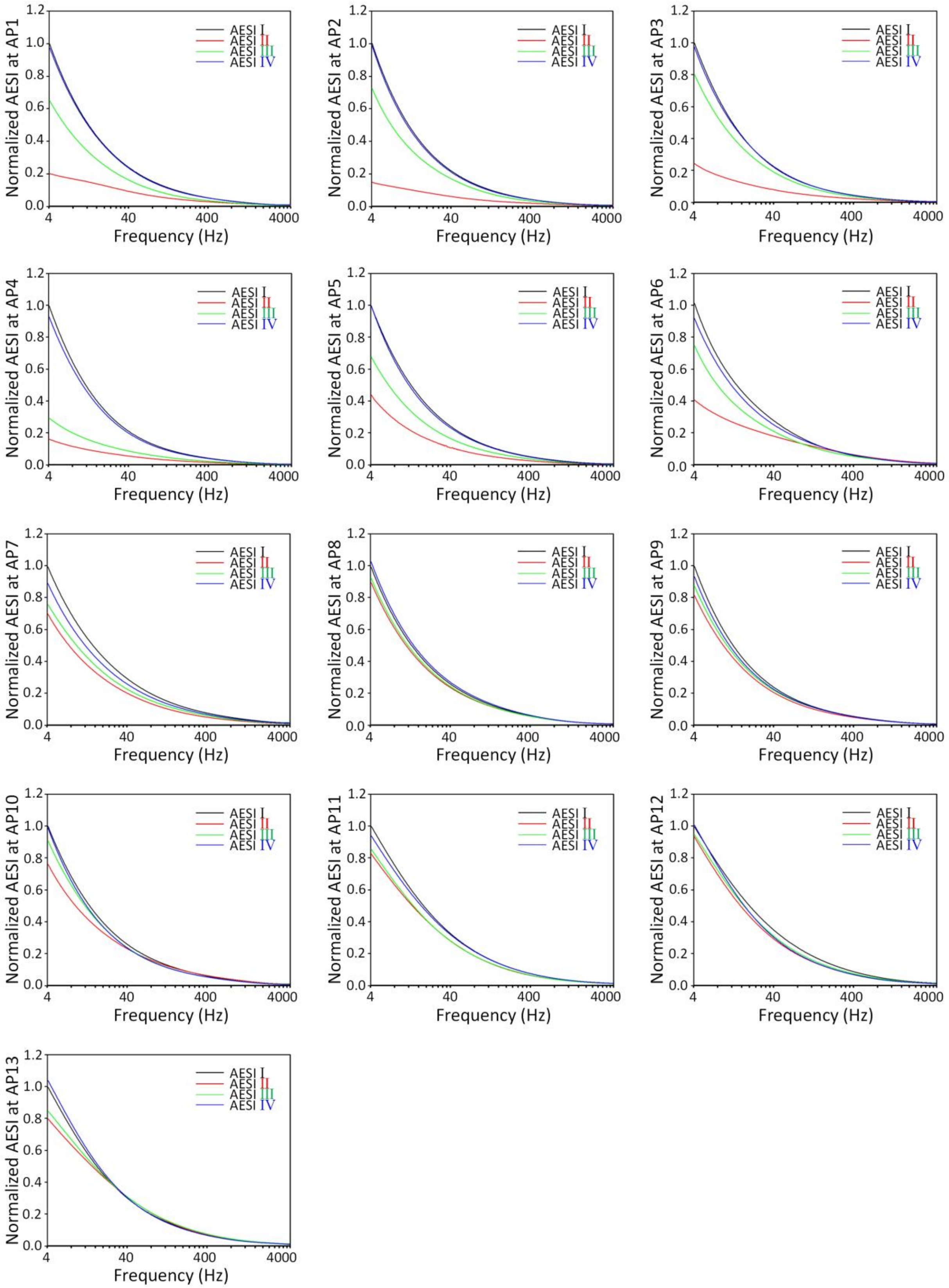

Figure S7. Normalized AESIs collected at AP1-13 across the four periods during cycling tests.

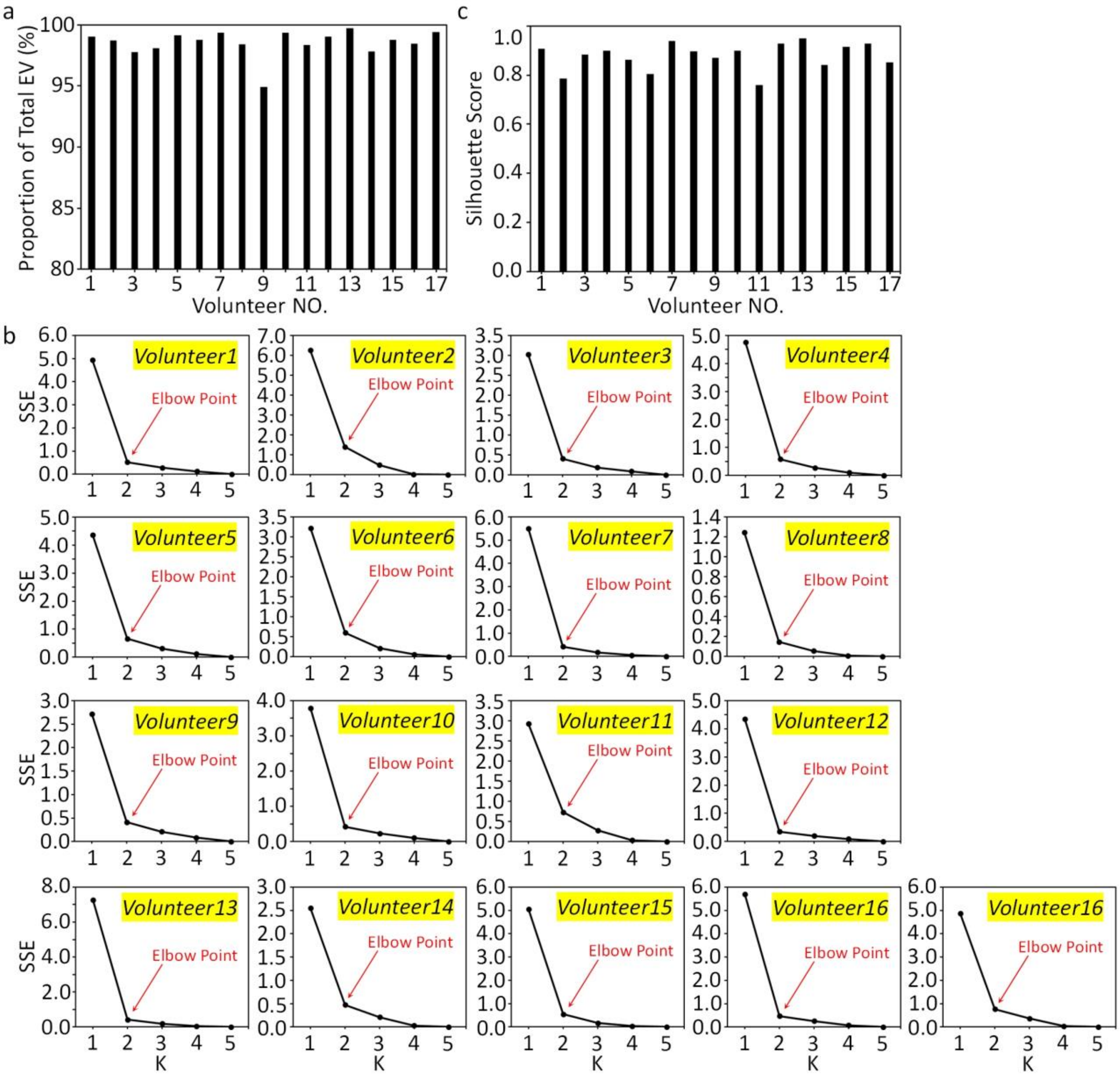
a
Proportion of Total EV (%)
Volunteer NO.
c
Silhouette Score
Volunteer NO.
b
SSE
K
Volunteer1
Volunteer2
Volunteer3
Volunteer4
Volunteer5
Volunteer6
Volunteer7
Volunteer8
Volunteer9
Volunteer10
Volunteer11
Volunteer12
Volunteer13
Volunteer14
Volunteer15
Volunteer16
Volunteer16
Elbow Point

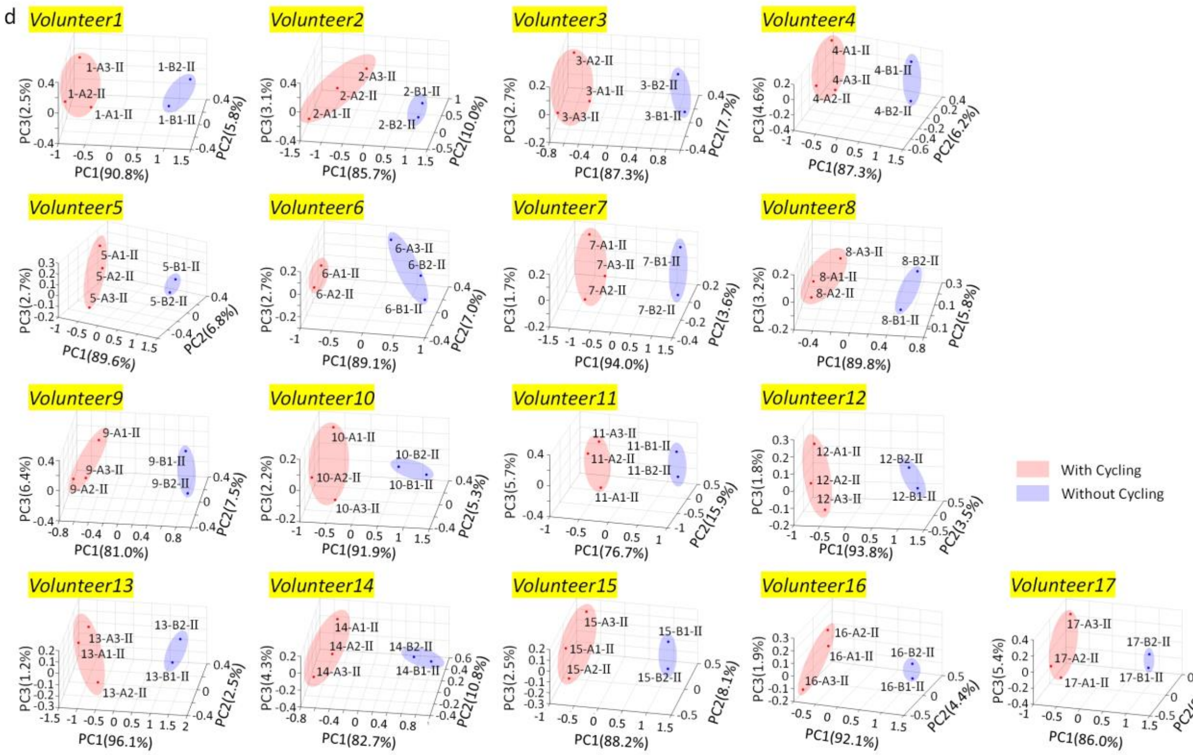

Figure. S8. Unsupervised machine learning results for the AESR datasets collected from the 17 individual volunteers in the cycling and control tests. **(a)** Column chart of the proportions of total explained variance from the raw datasets following PCA with the 3 PCs that explained the greatest amount of variance; **(b)** SSE changes with different values of cluster number K in the K-means clustering process; **(c)** Column chart of the S-scores in the K-means clustering process with K=2 for the datasets from all 17 volunteers ; **(d)** 3D scatter plots of the K-means clustering results with K=2 for the datasets from the 17 individual volunteers ("2-A1-II" denotes the AESR dataset collected in the period II in the first cycling test from volunteer 2).

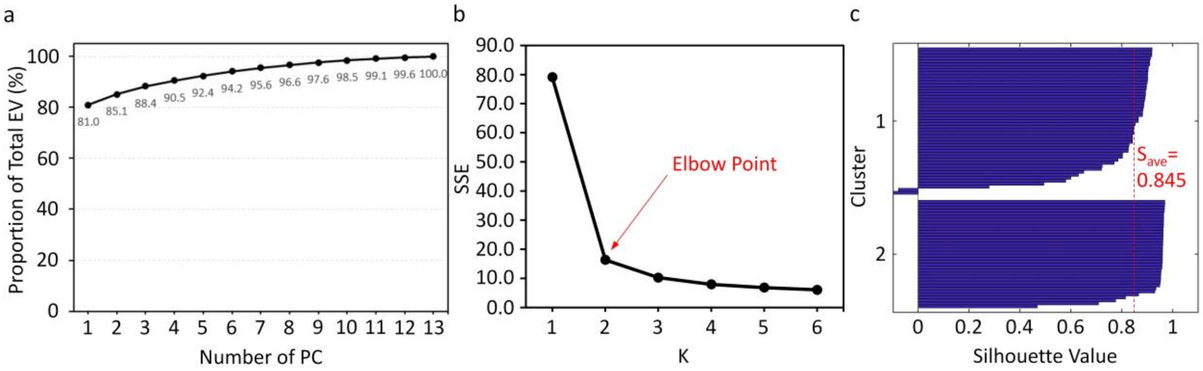

Figure. S9. Unsupervised machine learning results for the AESR datasets collected from all 17 volunteers in the cycling and control tests. **(a)** Proportions of total explained variance from the raw datasets following PCA with different numbers of PCs picked in order of decreasing variance contribution; **(b)** SSE changes with different values of cluster number K in the K-means clustering process; **(c)** S-score bar chart for the datasets in each cluster in the K-means clustering process with K=2.

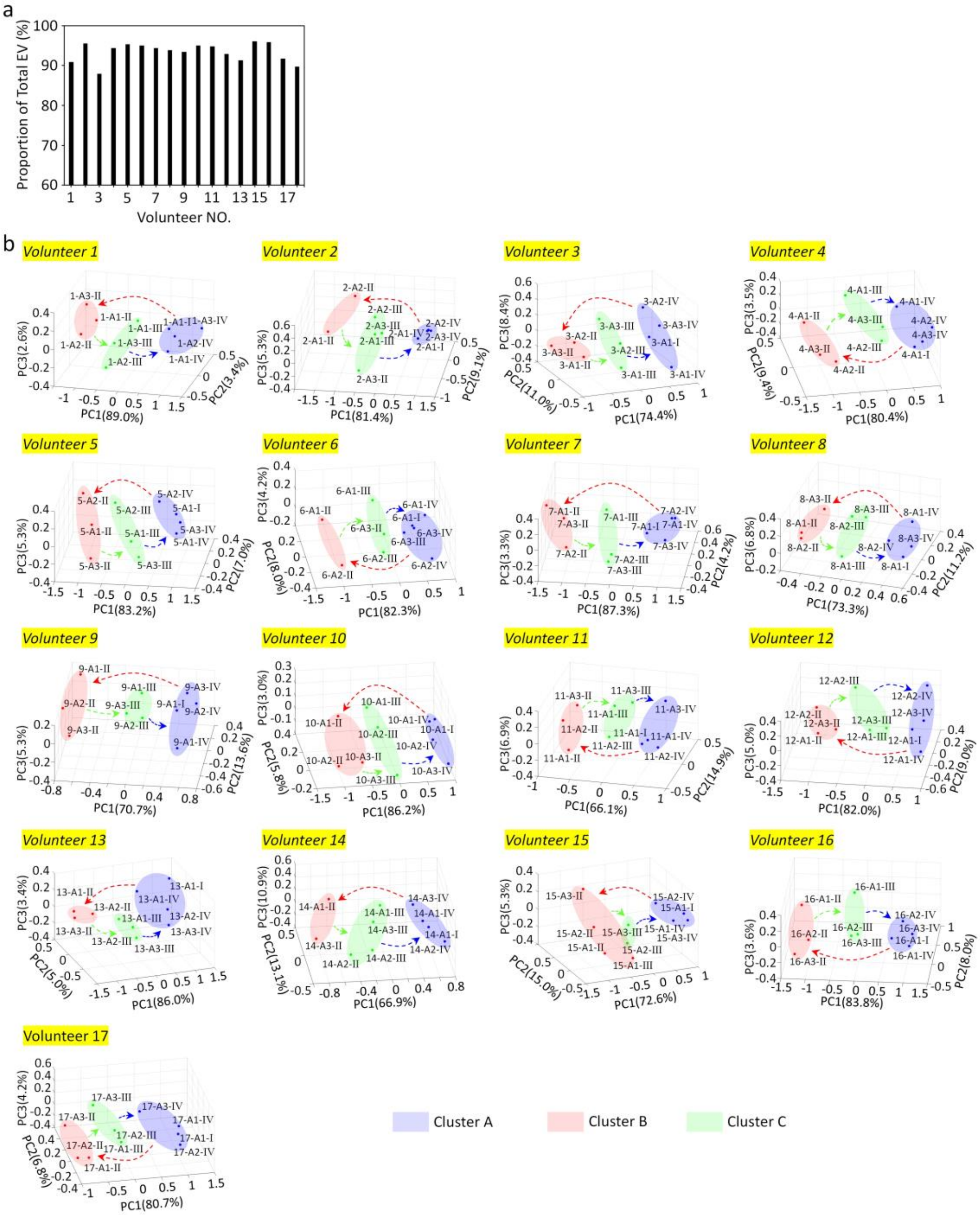

Figure. S10. Unsupervised machine learning results for the AESR datasets collected in all four periods in the cycling tests from the 17 individual volunteers. **(a)** Column chart of proportions of total explained variance from the raw datasets following PCA with the 3 PCs that explained the greatest amount of variance; **(b)** 3D scatter plots of the K-means clustering results with K=3 for the datasets from the 17 individual volunteers (“2-A1-II” denotes the AESR dataset collected in the period II in the first cycling test from volunteer 2).

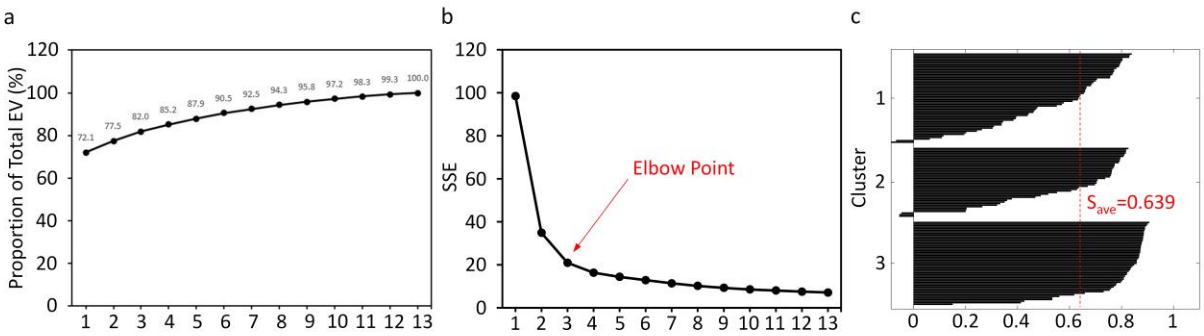

Figure. S11. Unsupervised machine learning results for the AESR datasets collected in all four periods in the cycling tests from all 17 volunteers. **(a)** Proportions of total explained variance from the raw datasets following PCA with different numbers of PCs picked in order of decreasing variance contribution; **(b)** SSE changes with different values of cluster number K in the K-means clustering process; **(c)** S-score bar chart for the datasets in each cluster in the K-means clustering process with K=3.

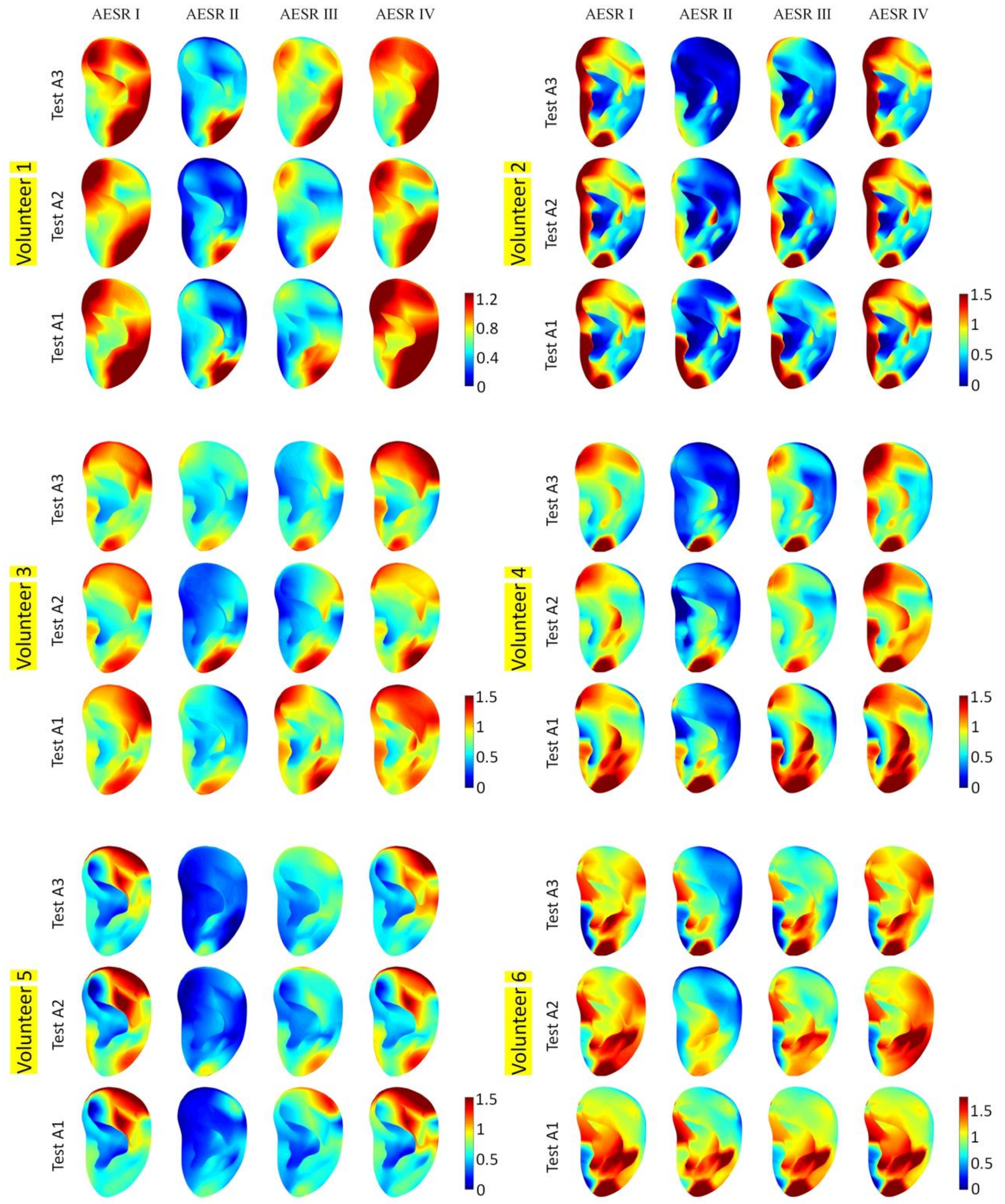

Figure. S12. 3D AESR contours change across the four periods in the cycling tests from the 17 individual volunteers. **(a)** volunteer 1-6;

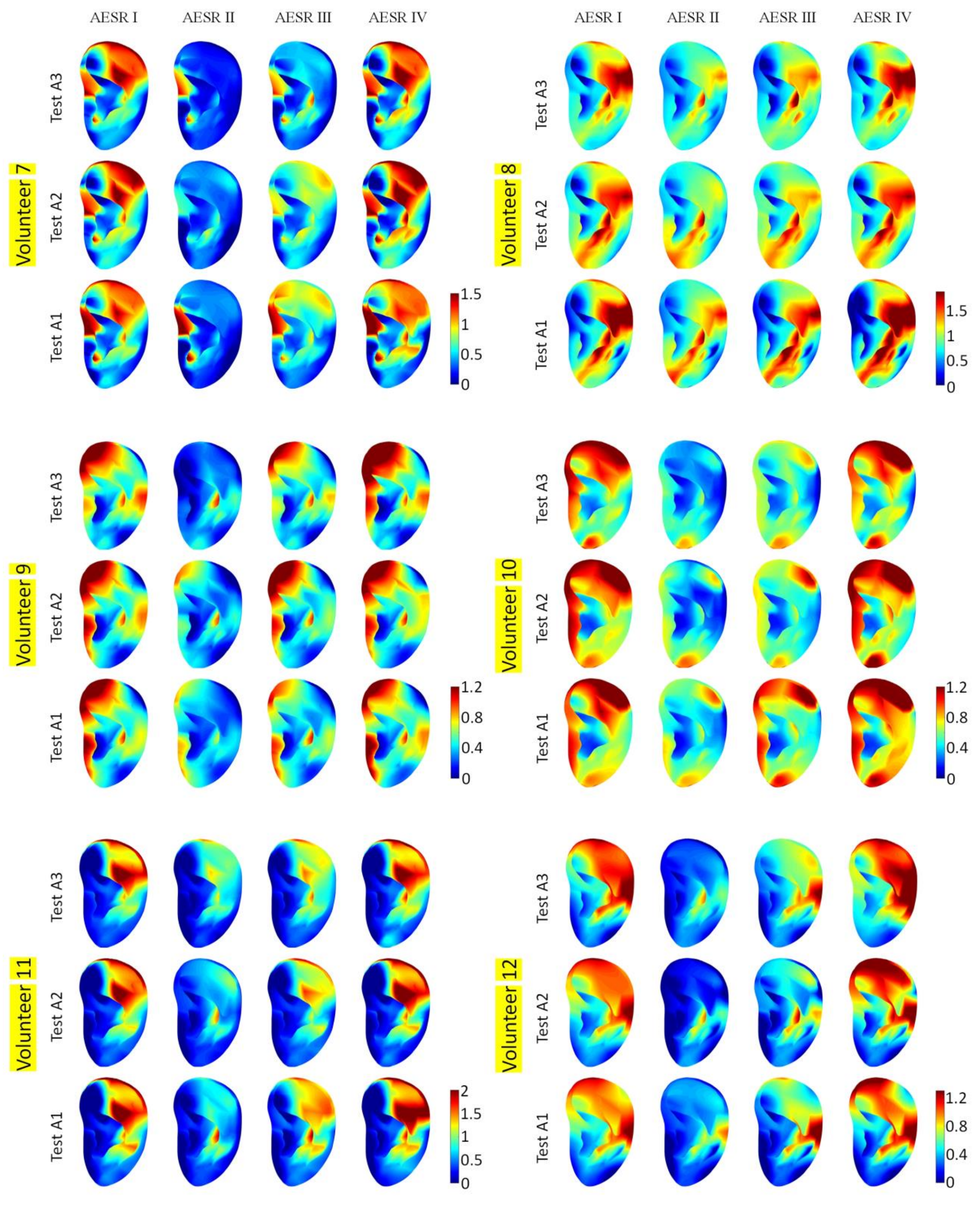

Figure. S12. 3D AESR contours change across the four periods in the cycling tests from the 17 individual volunteers. **(b)** volunteer 7-12;

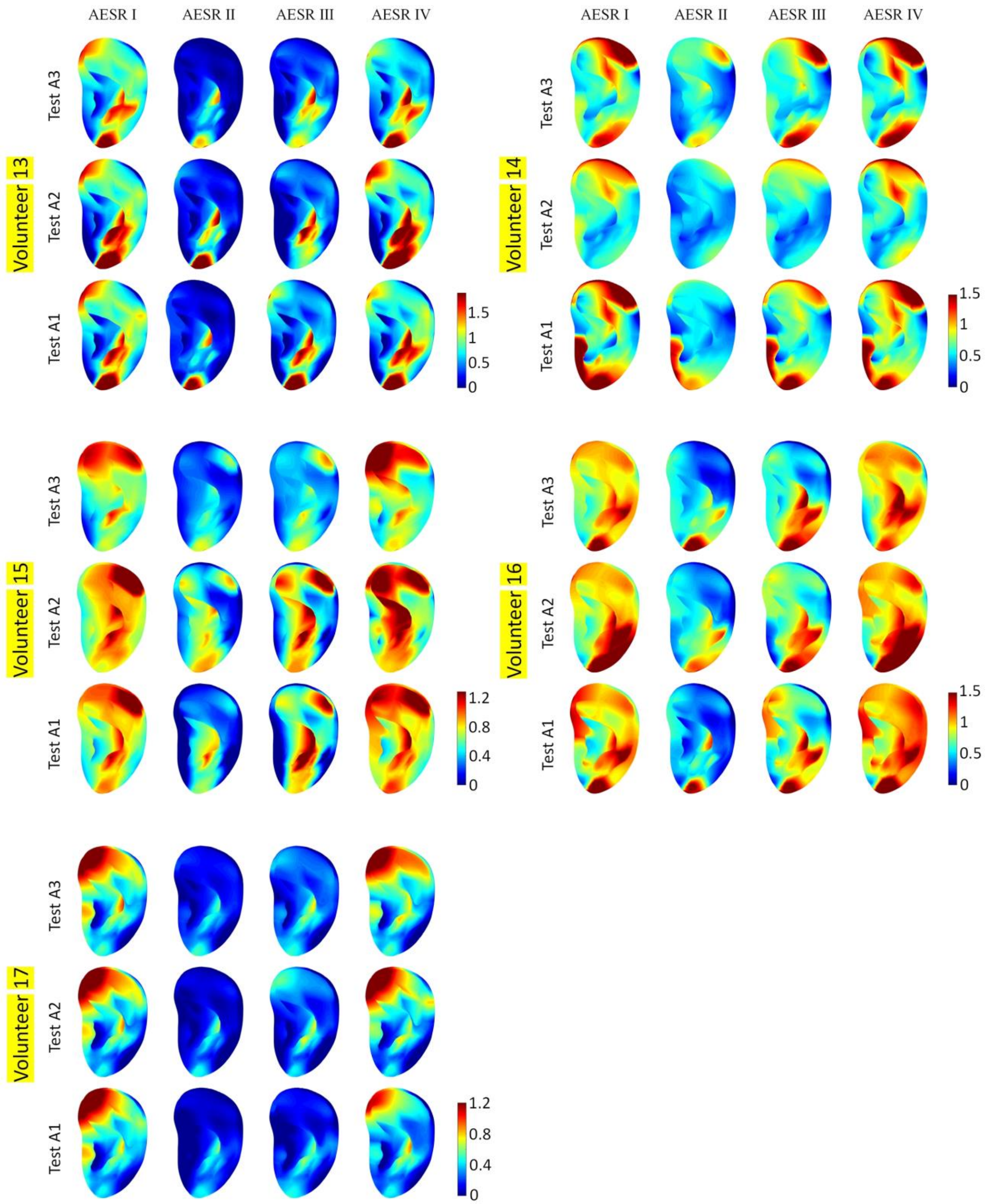

Figure. S12. 3D AESR contours change across the four periods in the cycling tests from the 17 individual volunteers. **(c)** volunteer 13-17.

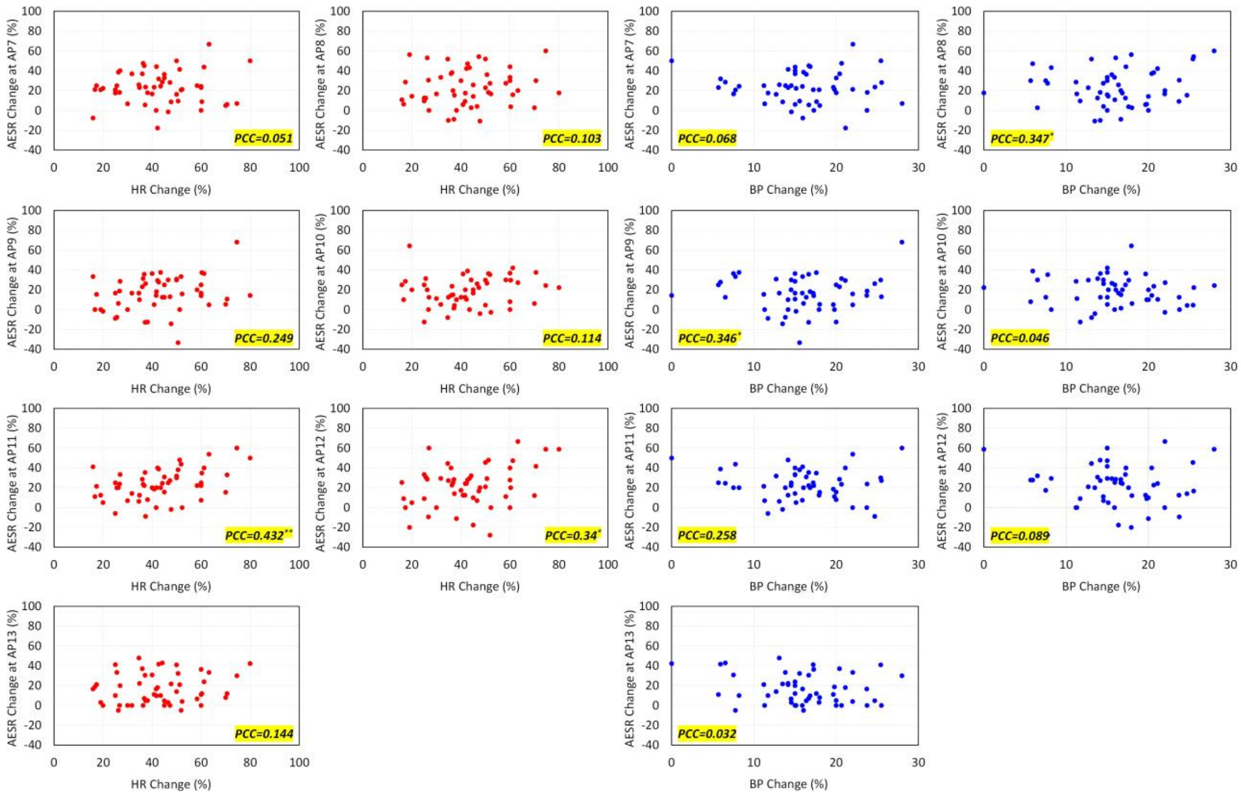

Figure. S13. Correlation analysis of AESR with HR and BP. Scatter plots of changes in AESR at AP7-13 versus changes in **(a)** HR and **(b)** BP after fixed-intensity cycling exercise (Biosignals II collected in all cycling tests from 17 volunteers are used).

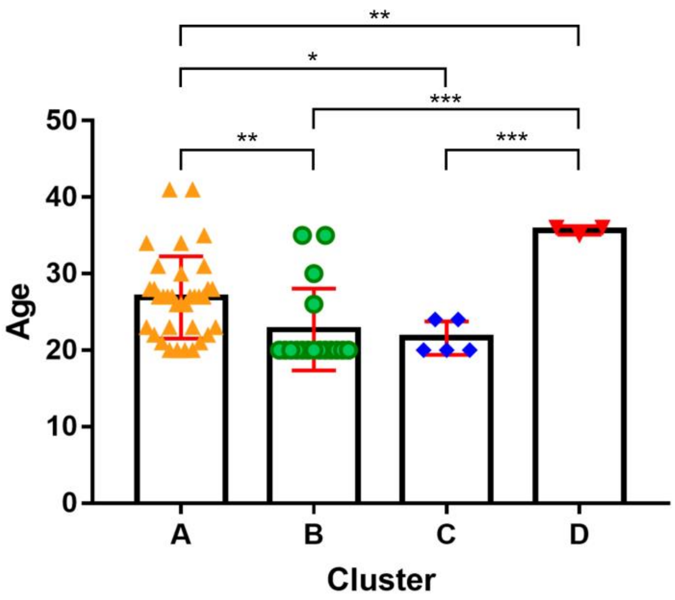

Figure. S14. Age statistical result by 4 clusters of the 30 human subjects (both right and left ears).

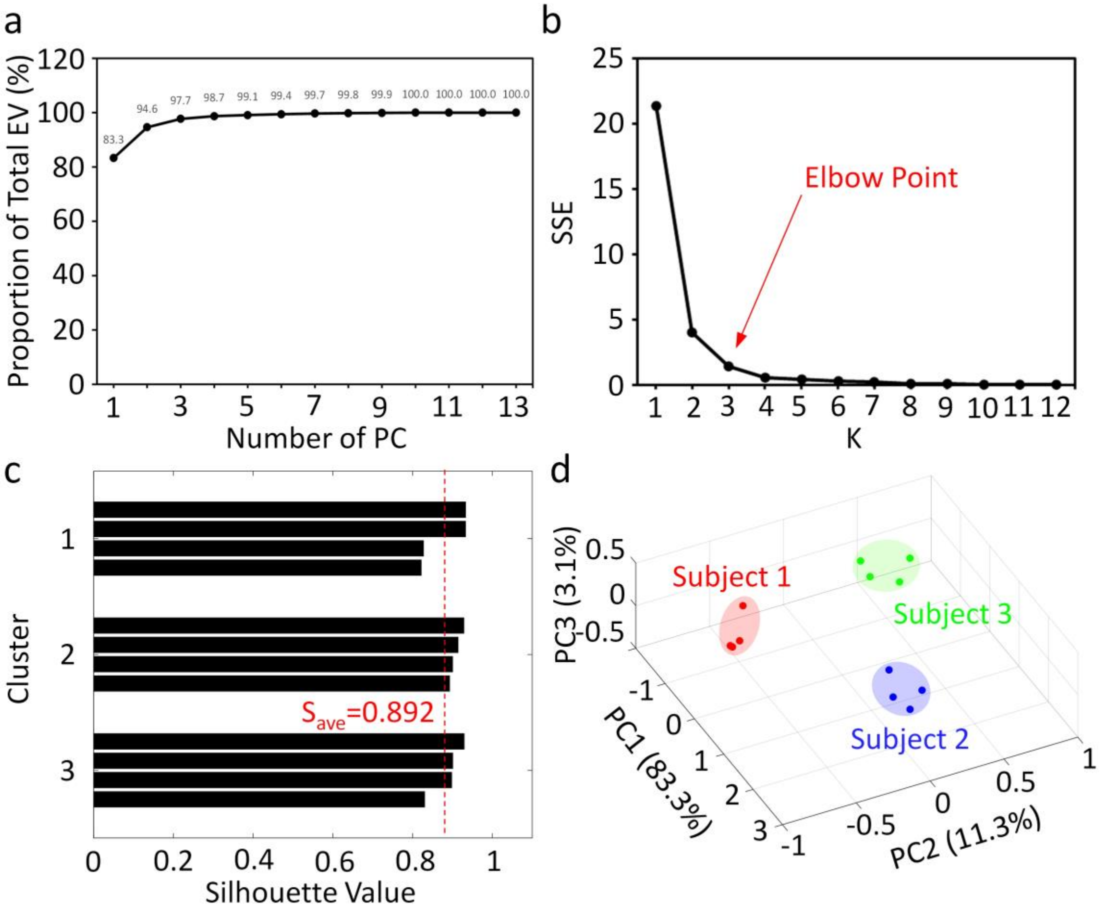

Figure. S15. Unsupervised machine learning results for the AESR datasets collected from 4 repeated measurements from 3 human subjects in cluster B. **(a)** Proportions of total explained variance from the raw datasets following PCA with different numbers of PCs picked in order of decreasing variance contribution; **(b)** SSE changes with different values of cluster number K in the K-means clustering process; **(c)** S-score bar chart for the datasets in each cluster in the K-means clustering process with K=3; **(d)** 3D scatter plot of the K-means clustering result with K=3 for the datasets from the 3 subjects in cluster B.

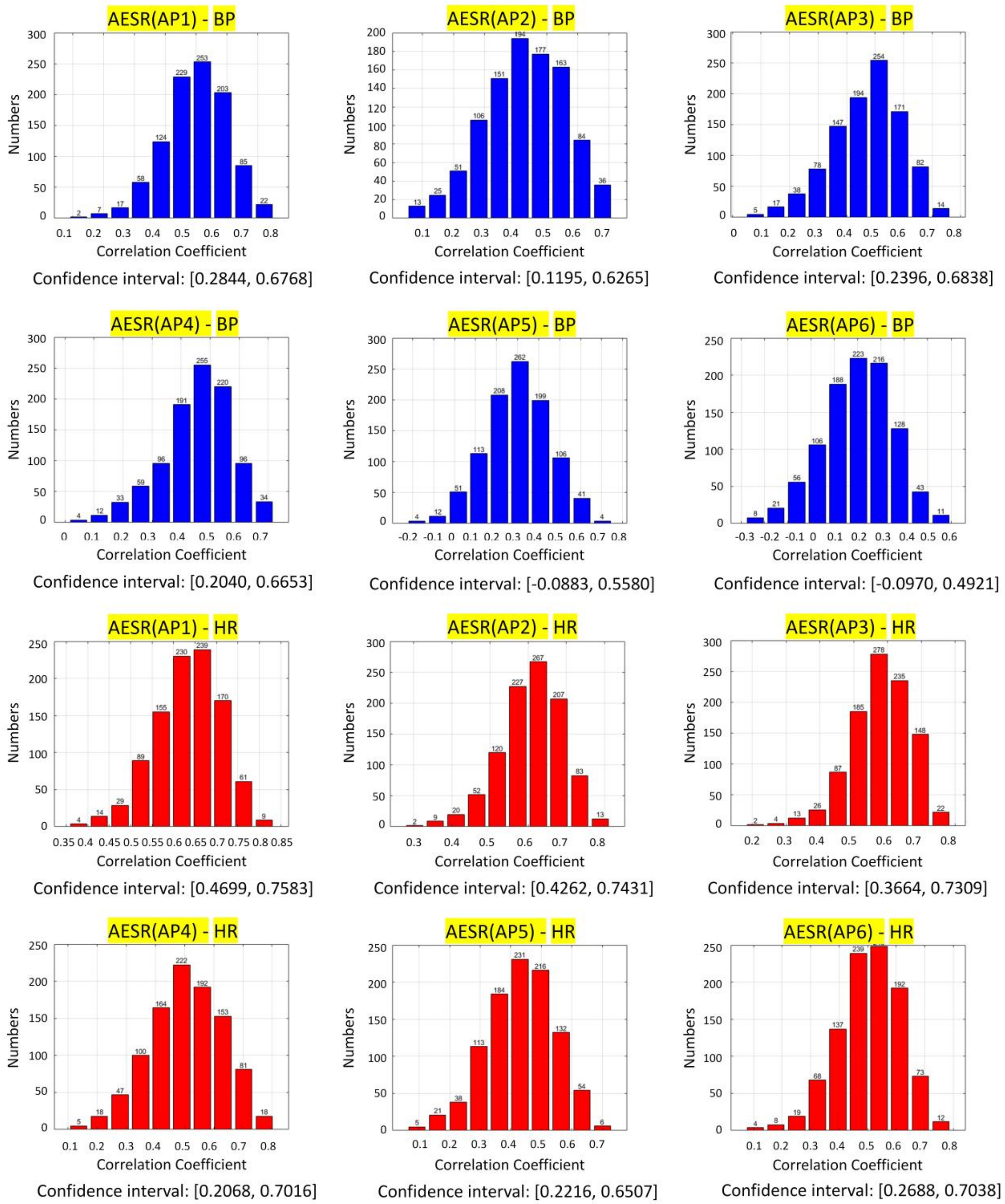

Figure. S16. Estimated correlation coefficient distribution histogram and 95% confidence intervals under Bootstrap analysis (sample size n = 1000).

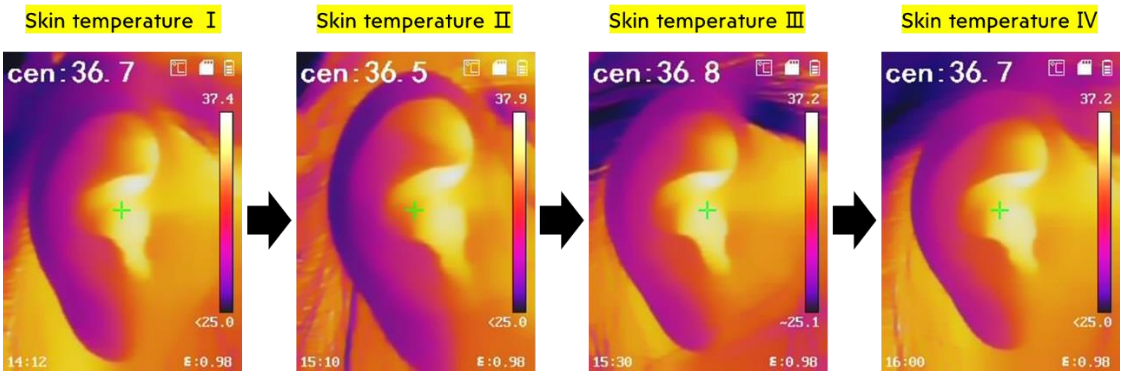

Figure. S17. Auricular skin temperature before and after stationary cycling.

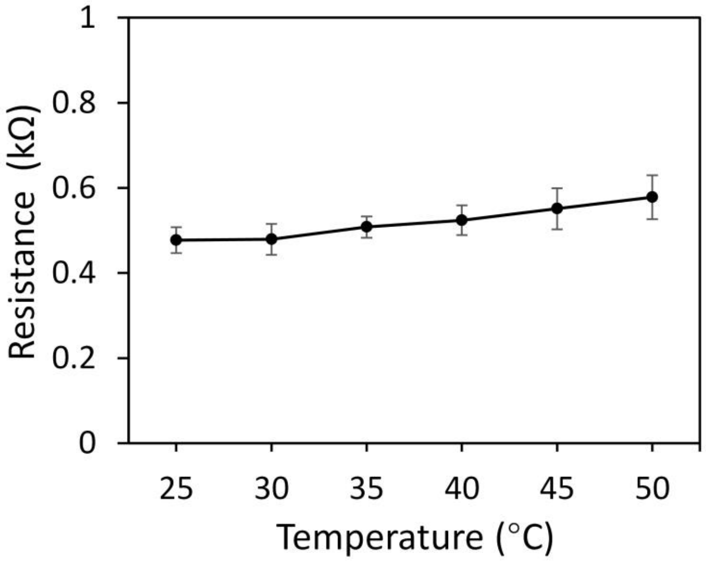

Figure. S18. Temperature coefficient of resistance measurement for the printable g-PLA filament (1.75 mm diameter, 15 mm length).

Printing Parameter	Setting Value
Infill Density	20%
Layer Height	0.1 mm
Printing Speed	50 mm/s
Extrusion Width	0.6 mm
Retraction Speed	40 mm/s
Z Hop at Retraction	0.2 mm/s
Outer Shell Wipe Speed	50 mm/s
Infill Speed	35mm/s
Infill Angle	0 deg
Maximum Increase Fan Speed	100%
Heated Bed Temperature	40 °C
Left Extruder (g-PLA)	190 °C
Right Extruder (TPE)	225 °C

Table. S1. Optimal printing parameters used for 3D-PAS prototyping.
